# Plasma Metabolome Normalization in Rheumatoid Arthritis Following Initiation of Methotrexate and the Identification of Metabolic Biomarkers of Efficacy

**DOI:** 10.3390/metabo11120824

**Published:** 2021-11-30

**Authors:** Matthew R. Medcalf, Pooja Bhadbhade, Ted R. Mikuls, James R. O’Dell, Rebekah L. Gundry, Ryan S. Funk

**Affiliations:** 1 Department of Pharmacy Practice, University of Kansas, Kansas City, KS 66160, USA; 2 Division of Allergy, Clinical Immunology and Rheumatology, Department of Internal Medicine, University of Kansas Medical Center, Kansas City, KS 66160, USA; pbhadbhade@kumc.edu; 3 Division of Rheumatology & Immunology, Department of Internal Medicine, University of Nebraska Medical Center (UNMC), Omaha, NE 68198, USA; tmikuls@unmc.edu (T.R.M.); jrodell@unmc.edu (J.R.O.); 4 VA Nebraska-Western IA Health Care System, Omaha, NE 68198, USA; 5 CardiOmics Program, Center for Heart and Vascular Research, Division of Cardiovascular Medicine, Department of Cellular and Integrative Physiology, University of Nebraska Medical Center, Omaha, NE 68198, USA; rebekah.gundry@unmc.edu; 6 Department of Pharmacology, Toxicology & Therapeutics, University of Kansas Medical Center, Kansas City, KS 66160, USA

**Keywords:** metabolomics, rheumatoid arthritis, methotrexate, biomarkers, plasma metabolome, metabolism

## Abstract

Methotrexate (MTX) efficacy in the treatment of rheumatoid arthritis (RA) is variable and unpredictable, resulting in a need to identify biomarkers to guide drug therapy. This study evaluates changes in the plasma metabolome associated with response to MTX in RA with the goal of understanding the metabolic basis for MTX efficacy towards the identification of potential metabolic biomarkers of MTX response. Plasma samples were collected from healthy control subjects (*n* = 20), and RA patients initiating MTX therapy (*n* = 20, 15 mg/week) before and after 16 weeks of treatment. The samples were analyzed by a semi-targeted metabolomic analysis, and then analyzed by univariate and multivariate methods, as well as an enrichment analysis. An MTX response was defined as a clinically significant reduction in the disease activity score in 28 joints (DAS-28) of greater than 1.2; achievement of clinical remission, defined as a DAS-28 < 2.6, was also utilized as an additional measure of response. In this study, RA is associated with an altered plasma metabolome that is normalized following initiation of MTX therapy. Metabolite classes found to be altered in RA and corrected by MTX therapy were diverse and included triglycerides (*p* = 1.1 × 10^−16^), fatty acids (*p* = 8.0 × 10^−12^), and ceramides (*p* = 9.8 × 10^−13^). Stratification based on responses to MTX identified various metabolites differentially impacted in responders and non-responders including glucosylceramides (GlcCer), phosphatidylcholines (PC), sphingomyelins (SM), phosphatidylethanolamines (PE), choline, inosine, hypoxanthine, guanosine, nicotinamide, and itaconic acid (*p* < 0.05). In conclusion, RA is associated with significant alterations to the plasma metabolome displaying at least partial normalization following 16 weeks of MTX therapy. Changes in multiple metabolites were found to be associated with MTX efficacy, including metabolites involved in fatty acid/lipid, nucleotide, and energy metabolism.

## 1. Introduction

Rheumatoid arthritis (RA) is an autoimmune inflammatory disease that primarily impacts joints but can affect a wide variety of tissues and organs [1]. Disease progression is associated with reduced life expectancy and chronic disability [2]. Current estimates range from 0.24 to 1.0% of the global population to be afflicted with RA [3]. To date, methotrexate (MTX) remains the cornerstone disease modifying antirheumatic drug (DMARD), due to its efficacy and economic merits [4]. MTX has been proven to slow the progression of RA while minimizing damage to tissues and joints. However, MTX therapy is often characterized by a highly variable response and a delayed onset of action. Approximately two-thirds of patients fail to have an adequate response to MTX therapy following six months of therapy [5]. The variable and unpredictable profile of MTX response has indicated a need for the identification of clinical biomarkers to guide drug therapy. To date, no reliable biomarkers exist that allow for the stratification of patients according to who will respond to MTX therapy or who will require alternative DMARDs (e.g., biologics) [6]. A major barrier to the identification of biomarkers of MTX response is an incomplete understanding of the molecular basis through which MTX mediates pharmacological effects in the treatment of RA [1].

Significant technological advances, and the advent of the ‘omics’ revolution, have made it possible to rapidly measure thousands of endogenous and exogenous low-molecular-weight molecules in a biological sample [7]. Metabolomics (i.e., the study of these metabolites) offers a relatively unbiased approach that can be applied to identify clinical biomarkers for early diagnosis, to stratify patient populations, to predict response to a given treatment, and to improve our understanding of metabolic changes associated with disease and therapeutic response [2,8]. Metabolomic methodologies examine the set of products and by-products of metabolic pathways, and thus can be used to identify specific drivers of biological processes, allowing us to better understand the physiological roles of specific metabolites [9]. Previous studies by our group have used metabolomics as a tool to understand biochemical changes occurring at the cellular level following initiation of MTX therapy, to identify metabolic pathways affected by MTX, and to identify metabolites and metabolic pathways that represent potential metabolic biomarkers of pharmacological response to MTX [6,10].

In this study, a semi-targeted metabolomic approach was used to identify changes in intermediates of primary metabolism, biogenic amines, and lipids in patients with RA. The metabolomic profiles of the respective cohorts were evaluated using chemometric and metabolic network enrichment analysis to determine differences in the plasma metabolome of patients with RA as compared with a healthy reference population, as well as determine the effects of MTX therapy on the plasma metabolome in RA. Then, stratification of RA patients based on clinical response to MTX was used to identify metabolites and metabolic pathways associated with the efficacy of MTX. The identified metabolites and metabolic pathways represent putative clinical biomarkers of MTX efficacy and further our understanding of the biochemical pharmacology of MTX in the treatment of RA.

## 2. Results

### 2.1. Patient Demographics and Clinical Data

Subjects in the study included a healthy control group (*n* = 20) and a prospective cohort of patients with RA initiating MTX therapy (*n* = 20). The entire cohort consisted of 40 subjects, 31 females (78%), with a median age of 54 (range of 21–84 years). RA patients in this study had a median of 11 months from symptoms to diagnosis and 2 months from diagnosis to the start of MTX at enrollment. Patients were excluded if they had previously received MTX; however, previous use of alternative conventional synthetic DMARDS were allowed, such as hydroxychloroquine. RA patients provided plasma samples at baseline, prior to initiating MTX, and 16 weeks following the initiation of MTX therapy. The demographic data of the cohorts are provided for comparison (Table 1) and did not differ significantly by age or gender. At week 16, following the initiation of MTX therapy, RA patients were characterized as “responders” and “non-responders” based on the change from baseline in their composite disease activity score in 28 joints (DAS-28). Those individuals that met the criteria of clinically significant improvement, a reduction in DAS-28 of greater than 1.2 (δDAS-28 (< −1.2)), were considered responsive to MTX therapy. An additional outcome of clinical remission was defined as achievement of a DAS-28 of less than 2.6 (DAS-28 < 2.6) following 16 weeks of MTX therapy. As expected, measures of disease activity were significantly lower at 16 weeks post MTX therapy for those found to be responsive to MTX therapy including swollen joint count (SJC), tender joint count (TJC), global health assessment, and DAS-28. Among the 11 responders to MTX therapy according to a reduction in DAS-28 of greater than 1.2, 8 (73%) patients were found to reach clinical remission. However, ESR was not found to differ significantly based on response.

### 2.2. Changes in the Plasma Metabolome Associated with RA and the Effect of MTX Therapy

**Figure 1:**
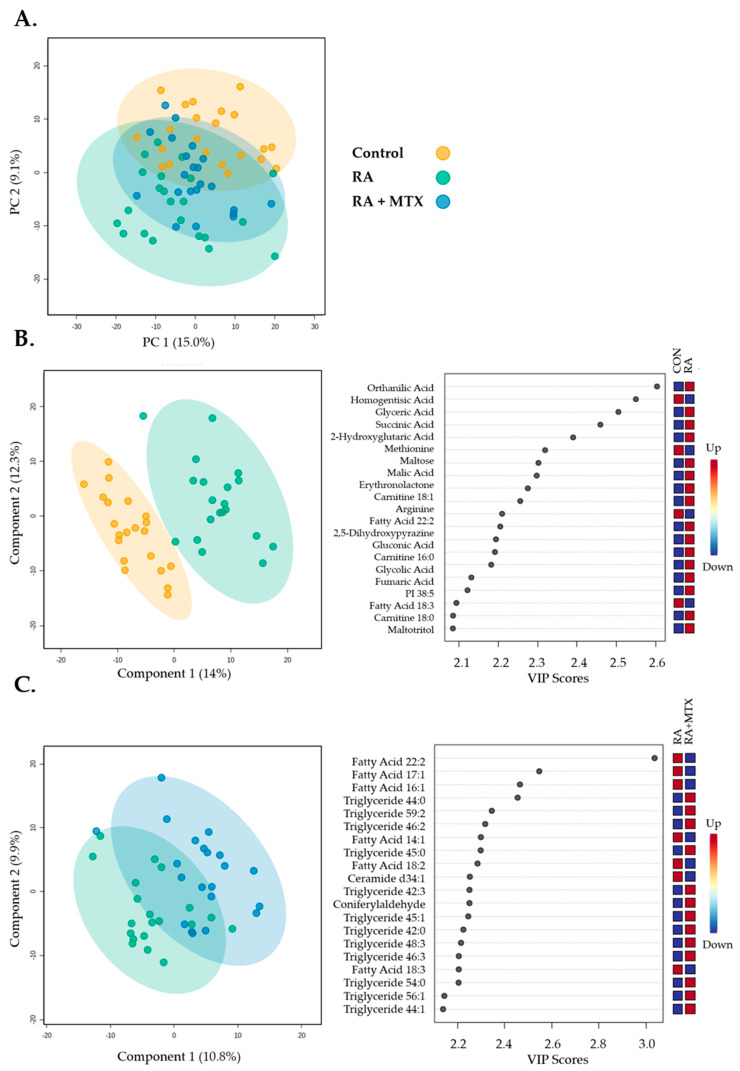
Multivariate unsupervised and supervised analysis of 647 metabolites identified from individual patient samples providing 2D score plots displaying principal components within the healthy control subjects (control, yellow), RA patients at baseline (RA, green), and following 16 weeks of MTX therapy in RA patients (RA + MTX, blue): (**A**) Principal components analysis (PCA); (**B**) partial least squares discriminant analysis (PLS-DA) and variable importance plots (VIP) between healthy control subjects and RA patients at baseline (R^2^ = 0.99 and Q^2^ = 0.68); (**C**) partial least squares discriminant analysis (PLS-DA) and variable importance in projection (VIP) plots between RA patients at baseline and following 16 weeks of MTX therapy (R^2^ = 0.97 and Q^2^ = −0.69).

The plasma metabolomic data included 647 identified metabolites that were analyzed using an unpaired multivariate analysis and visualized using principal components analysis (PCA) (Figure 1A). PCA was chosen as an exploratory and unsupervised analysis to reduce the dimensionality of the total metabolomics dataset in order to evaluate differences in the metabolome between healthy control subjects and patients with RA, both at baseline and 16 weeks after initiation of MTX. On the basis of 95% confidence intervals, the plasma metabolome of healthy subjects displayed a reasonable separation from RA patients at baseline. At 16 weeks post MTX therapy, the metabolome of patients with RA displayed an increased overlap with healthy subjects. Albeit incomplete, this suggests a normalization of the plasma metabolome following the initiation of MTX therapy.

Then, the plasma metabolomic data were submitted to a supervised multivariate analysis and visualized using a partial least squares discriminant analysis (PLS-DA) for the initial identification of metabolites of interest. The plasma metabolomic data from healthy control subjects and RA patients at baseline displayed reasonable separation, appropriate fitting of the data, and provided a variable importance plot (VIP) (Figure 1B, R^2^ = 0.99, and Q^2^ = 0.68). Similarly, plasma metabolomic data from a paired analysis of RA patients at baseline and following 16 weeks of MTX therapy were submitted to PLS-DA, providing PLS-DA and VIP (Figure 1C, R^2^ = 0.97, and Q^2^ = −0.69). While the VIP was able to identify top differentiating metabolites, the overfitting of the data should be noted.

**Figure 2:**
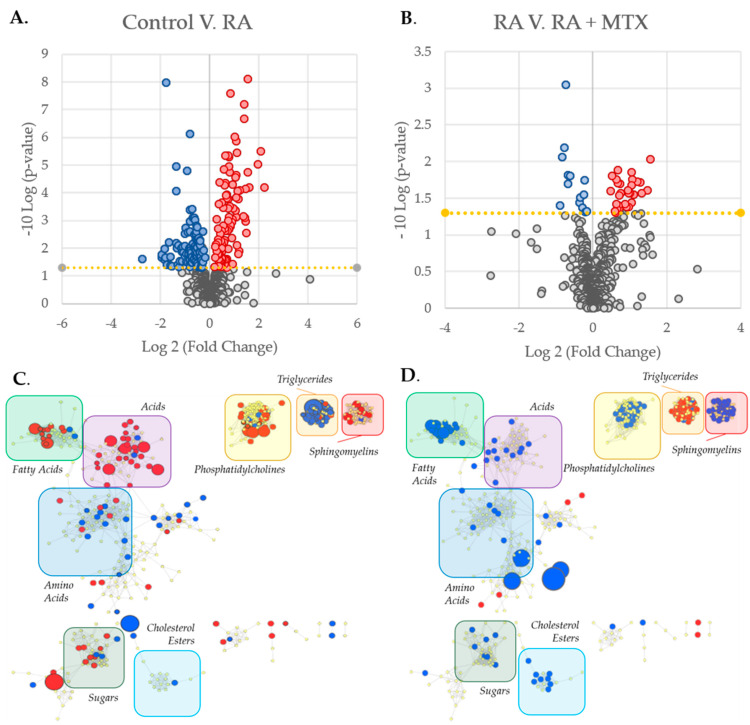
Identification and metabolic network mapping of key metabolites associated with RA and MTX therapy. Metabolomics data were analyzed with MetaboAnalyst 5.0 resulting in volcano plots for: (**A**) Healthy control subjects as compared with RA patients at baseline; (**B**) RA patients at baseline as compared with 16 weeks following the initiation of MTX. The metabolic network maps were built using MetaMapp 2020 and visualized using Cytoscape 3.7.2 providing maps of: (**C**) Healthy control subjects as compared with RA patients at baseline; (**D**) RA patients at baseline as compared with 16 weeks after the initiation of MTX. The metabolomic networks were divided into clusters using the community cluster tool and labeled by metabolic class. Red denotes metabolites found to be increased and blue denotes metabolites found to be decreased (*p* < 0.05). Node size is directly proportional to the measured fold change.

**Figure 3:**
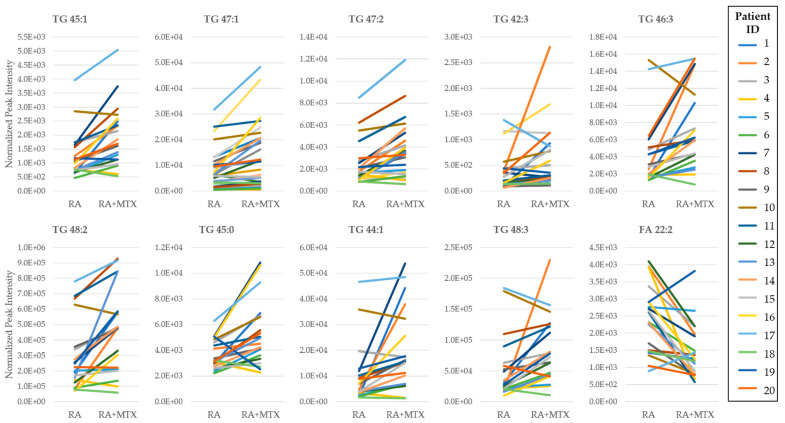
Pair-wise comparison of the top ten ranking metabolites identified from the volcano plot of the plasma metabolome of RA patients at baseline and following 16 weeks of MTX therapy. Rankings were based on *p*-value. Normalized peak intensities for each RA patient at baseline (RA) and following 16 weeks of MTX therapy (RA + MTX). Triglyceride (TG); fatty acid (FA).

The identification of individual metabolites altered in patients with RA was based on a comparison of differences in metabolite levels from plasma collected from healthy control subjects and RA patients prior to initiation of MTX (i.e., at baseline) (Figure 2A). Similarly, identification of metabolites associated with MTX therapy in RA was based on a paired analysis of metabolite levels in RA patients at baseline and following 16 weeks of MTX therapy (Figure 2B). Those metabolites that met the *p*-value threshold of less than 0.05 were deemed to be metabolites of interest for subsequent analysis. On the basis of this threshold, 214 metabolites were identified to differentiate healthy subjects and patients with RA (Table S1), and 44 metabolites were found to be altered in RA patients following treatment with MTX (Table S2). Metabolites identified as top discriminating metabolites by univariate analysis widely overlapped with those identified by PLS-DA. On the basis of the *p*-value, the top ten ranking metabolites identified by comparing RA patients to healthy control subjects at baseline included increases in orthanilic acid, glyceric acid, succinic acid, 2-hydroxyglutaric acid, malic acid, erythronolactone, CAR 18:1, and FA 22:2, as well as decreases in homogentisic acid and methionine. The top ten metabolites altered following initiation of MTX therapy included increases in saturated and unsaturated triglycerides (TG 45:1, TG 47:1, TG 47:2, TG 42:3, TG 46:3, TG 48:2, TG 45:0, TG 44:1, and TG 48:3) and a decrease in fatty acids (FA 22:2). Next, metabolite levels for the top ten metabolites altered following 16 weeks of MTX therapy were plotted to allow for visual comparison of metabolite levels for individual patients (*n* = 20) (Figure 3). The overall trends observed at the individual patient level correspond with the data in the volcano plots; however, these data also demonstrate significant inter-patient variability in both the abundance of the metabolites and their responsiveness to 16 weeks of MTX therapy.

To better identify the relationships between metabolites of interest, a metabolic network map highlighting the differences between patients with RA and healthy control subjects was built using MetaMapp 2020 (University of California, Davis; Davis, CA, USA) and visualized in Cytoscape (Version 3.7.2, Institute for Systems Biology, Seattle, Washington, USA) (Figure 2C). The network map was divided into clusters and labeled by classes of metabolites indicating several changes in the metabolome in RA relative to controls, including increases in fatty acids, sphingomyelins, and phosphatidylcholines, as well as decreases in triglycerides and amino acids. Similarly, a metabolic network map was built to highlight changes in the plasma metabolome following 16 weeks of MTX therapy in patients with RA (Figure 2D). The network map highlights several changes to the metabolome in response to MTX therapy, including increases in triglycerides and decreases in fatty acids, sphingomyelins, phosphatidylcholines, and cholesterol esters. The decreases observed in fatty acids, sphingomyelins, and phosphatidylcholines, as well as the increase in triglycerides found to be associated with MTX therapy all correlate with a correction towards the plasma metabolome observed in healthy control subjects.

### 2.3. Enrichment Analysis to Identify Metabolite Classes Associated with RA and the Impact of MTX

**Figure 4:**
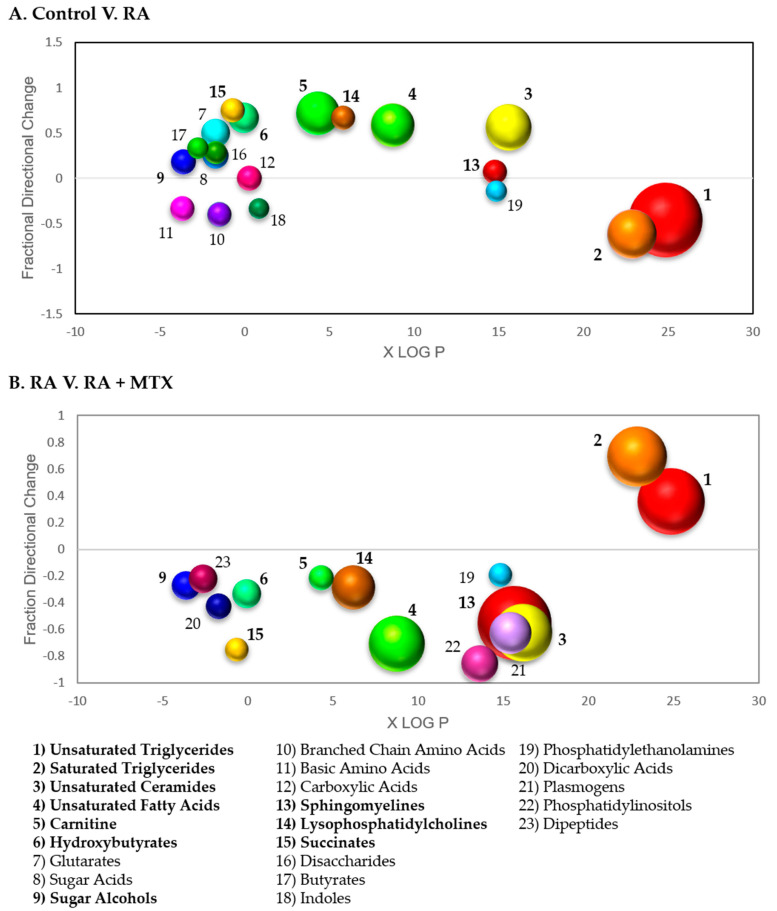
Chemometric enrichment analysis of metabolomic profiling data: (**A**) Metabolomic data comparing RA patients at baseline to healthy control subjects was analyzed using ChemRICH open-source software to produce nonoverlapping chemical cluster classifications mapping 551 of the total 647 metabolites to 57 nonoverlapping chemical clusters. Among these clusters differentiating healthy control subjects and RA patients, 19 clusters were found to be statistically significant using an FDR-adjusted *p*-value cutoff of 0.05; (**B**) metabolomic data from RA patients at baseline and following 16 weeks of MTX therapy was analyzed and 551 of the total 647 metabolites mapped to 56 nonoverlapping chemical clusters. Among these clusters differentiating baseline disease state and sixteen weeks post MTX therapy, 19 clusters were found to be statistically significant. Each cluster of metabolites was plotted based on lipophilicity, fraction directional change, and *p*-value; node size is directly proportional to the negative logarithm of the *p*-value for each cluster. Bolded clusters of metabolites were found to display a correction towards levels observed in healthy control subjects following the initiation of MTX in RA patients.

In order to further analyze and visualize the data, differences in the measured metabolites associated with RA were subjected to a chemometric enrichment analysis using the open-source software ChemRICH. A total of 57 nonoverlapping chemical clusters were identified, with 19 of these chemical clusters found to be significantly altered in RA based upon an FDR-adjusted *p*-value cutoff of 0.05 (Table S3). Then, metabolite clusters found to be significant were plotted according to fraction directional change and median xlogP for the given cluster (Figure 4A). The fraction directional change was calculated based on the net directional change in metabolite clusters divided by the total number of metabolites measured in each cluster. For example, sphingomyelins were found to be altered in RA with 3 metabolites found to be increased, and 1 metabolite found to be decreased of the total 29 metabolites in the cluster measured, resulting in a fraction directional change of 0.07 (i.e., (3−1)/29 = 0.07). Node size is directly proportional to the negative log *p*-value for each cluster of metabolites. Significant changes were observed in lipid metabolism, with decreases in both saturated (*p* = 4.3 x 10^−10^) and unsaturated triglycerides (*p* = 2.2 × 10^−20^) and increases in unsaturated fatty acids (*p* = 6.2 × 10^−8^). In addition, increases in clusters of metabolites for both unsaturated ceramides (*p* = 7.0 × 10^−9^) and carnitines (*p* = 6.6 × 10^−8^) were observed.

Chemometric enrichment analysis of metabolic changes observed following the initiation of MTX in patients with RA afforded a total of 56 nonoverlapping chemical classes, with 19 of these chemical classes found to be altered significantly based on an FDR-adjusted *p*-value cutoff of 0.05 (Table S4). A plot of fraction directional change/difference and median xlogP demonstrates a correction of several metabolite clusters in association with MTX therapy, with increases in both saturated (*p* = 1.3 × 10^−13^) and unsaturated (*p* = 1.1 × 10^−16^) triglycerides, and a decrease in saturated (*p* = 0.00027) and unsaturated (*p* = 8.0 × 10^−12^) fatty acids (Figure 4B). In addition, decreases were observed in unsaturated phospholipids (*p* = 5.3 × 10^−14^), unsaturated sphingomyelins (*p* = 2.2 × 10^−20^), cholesterol esters (*p* = 9.8 × 10^−8^), and unsaturated lysophosphatidylcholines (*p* = 9.5 × 10^−8^).

### 2.4. Identification of Metabolites Altered in RA Are Corrected Following Initiation of MTX

**Figure 5:**
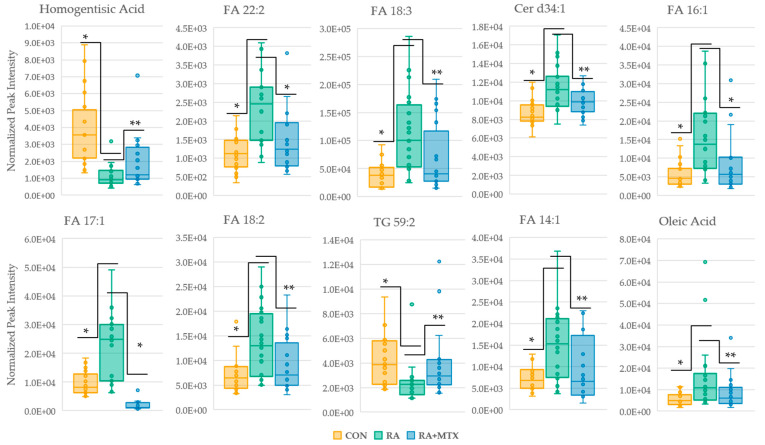
Box and whisker plots of statistically significant metabolites (* = *p* < 0.005 and ** = *p* < 0.05) identified in volcano plots displaying a correction towards metabolite levels of healthy control. Control (yellow); RA (green); RA + MTX (blue); fatty acid (FA); ceramide (Cer); triglyceride (TG).

Plasma levels of metabolites of interest (*p* < 0.05) from the volcano plots identified a total of 35 overlapping metabolites that were altered in RA and displayed at least a partial correction towards healthy control levels following 16 weeks of MTX therapy (Table S6). The top ten metabolites found to display a correction towards healthy control levels ranked according to *p*-value were plotted as box and whisker plots (Figure 5). A total of 10 (29%) metabolites identified were found to increase in active RA and decrease following MTX therapy. These metabolites include primarily fatty acids (*n* = 7), as well as ceramides (*n* = 3). A total of 25 (71%) metabolites were identified to decrease in active RA and increase following MTX therapy. These metabolites include primarily triglycerides (96%). Among the 35 statistically significant metabolites identified, 34 (97%) metabolites are associated with fatty acid/lipid metabolism.

To further identify metabolites associated with disease activity in RA, all metabolites were submitted to a Spearman’s regression analysis to define the correlation between DAS-28 score and metabolite levels at baseline and 16 weeks after initiating MTX therapy (Table S5). The analysis identified 36 statistically significant metabolites (*p* < 0.05) that correlate with DAS-28. Approximately 90% of the identified metabolites are associated with fatty acid/lipid metabolism, including predominantly saturated and unsaturated triglycerides. Triglycerides identified by Spearman’s regression analysis to correlate inversely with DAS-28 score were shown to increase following MTX therapy, displaying a correction towards levels found in the healthy control group.

### 2.5. Identification of Plasma Metabolites Associated with MTX Response after 16 Weeks of Treatment

**Figure 6:**
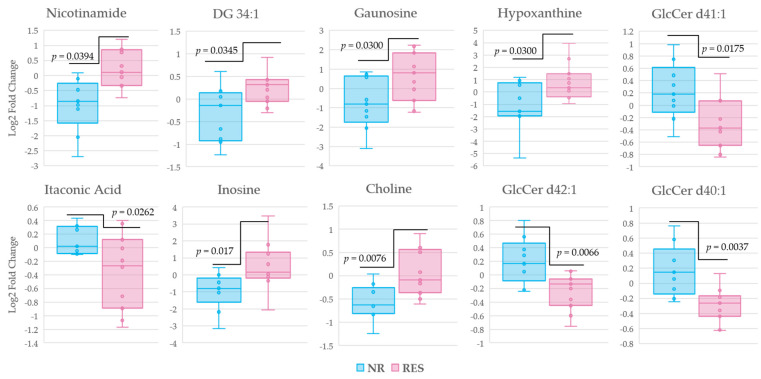
Box and whisker plots of changes in metabolite levels distinguishing non-responders and responders according to a δDAS (−1.2), representing a lowering in disease activity score of 1.2. Then, metabolites were cross referenced according to DAS-28 < 2.6, the threshold recognized as remission in RA. The log normalized change in peak intensity for ten metabolites from baseline to sixteen weeks post MTX therapy identified by univariate analysis are displayed. Non-responders (blue); responders (pink); diglyceride (DG); glucosylceramide (GlcCer).

In order to identify metabolic changes and metabolites associated with response to MTX therapy, changes in metabolite levels across the 16 week MTX treatment period were compared between patients identified as responders and non-responders according to δDAS-28 (−1.2). A total of 19 metabolites associated with response to MTX therapy were found to be statistically significant (*p* < 0.05). These metabolites were further assessed for their relationship to response to MTX therapy based upon achievement of clinical remission (i.e., DAS-28 < 2.6) [11]. Those metabolites identified to differentiate responders and non-responders by both thresholds of δDAS-28 (−1.2) and DAS-28 < 2.6 were plotted according to log normalized change in peak intensity (Figure 6). Statistically significant (*p* < 0.05) relative increases were observed in choline, inosine, hypoxanthine, guanosine, nicotinamide, and diglyceride (DG) 34:1 in patients found to be responsive to MTX therapy, while relative decreases were observed in glucosylceramide (GlcCer) d40:1, GlcCer d42:1, GlcCer d41:1, and itaconic acid.

**Figure 7:**
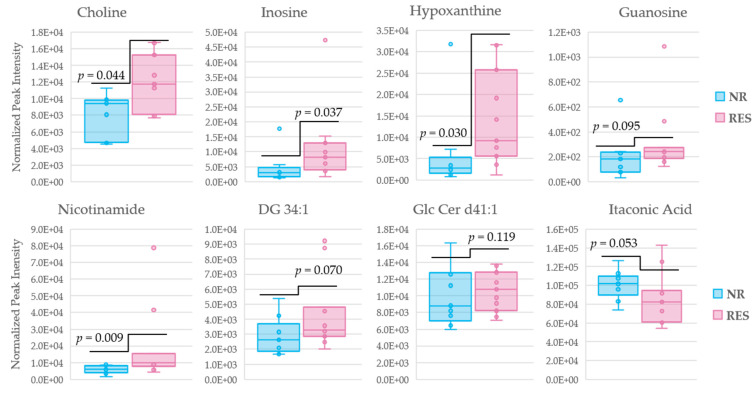
Box and whisker plots of metabolite levels after 16 weeks of MTX therapy distinguishing non-responders and responders according to a δDAS (−1.2), representing a lowering in disease activity score of 1.2. The log normalized peak intensities for the eight metabolites identified by AUC analysis. Non-responders (blue); responders (red); diglyceride (DG); glucosylceramide (GlcCer).

To further access the roles of the discriminating metabolites identified above in the pharmacological response to MTX, as well as their roles as potential therapeutic biomarkers, receiver operator characteristic (ROC) curves were generated for the identified metabolites. Patients were first stratified into responders and non-responders in accordance with an improvement in DAS-28 of more than 1.2. The ROC curves were generated from metabolite levels measured at week 16 of MTX therapy (Table S7). Metabolites previously identified to differentiate responders and non-responders following 16 weeks of MTX therapy with an area under the curve (AUC) greater than 0.70 include choline (AUC = 0.77), inosine (AUC = 0.78), hypoxanthine (AUC = 0.79), guanosine (AUC = 0.73), nicotinamide (AUC = 0.85), and DG 34:1 (AUC = 0.74), GlcCer d41:1 (AUC = 0.71), and itaconic acid (AUC = 0.76). The log normalized peak intensities of metabolites that meet the threshold of an AUC greater than 0.70 following 16 weeks of MTX therapy were then plotted (Figure 7). Among the eight metabolites identified, five displayed a *p*-value < 0.05 in discriminating responders and non-responders after MTX therapy.

## 3. Discussion

In this study, changes to the plasma metabolome associated with RA and the effect of MTX therapy were first identified. A robust change occurs in the metabolite levels of primary metabolism, biogenic amines, and lipids in both active RA compared with healthy control subjects and between active RA patients both before and after 16 weeks of MTX therapy (Figure 2C,D). Discriminating metabolites were identified by both multivariate and univariate statistical analyses and yielded overlapping results. Top discriminating metabolites for the comparison of RA patients with healthy control subjects identified alterations in organic acid metabolism with a shift towards an increase in various organic acids in the plasma of patients with RA. By comparison the plasma metabolome at baseline and 16 weeks in the RA patient’s results yielded fatty acids and triglycerides as the top discriminating metabolites and supported a reduction in fatty acid levels and a corresponding increase in triglycerides in RA patients following the initiation of MTX. The chemical enrichment analysis further supported significant changes to a variety of metabolic classes involved in fatty acid metabolism in RA that were corrected upon MTX therapy. Further changes in metabolite levels identified via pathway analysis reflect the biochemical impact of MTX. Pathways of particular interest include purine and energy metabolism. After characterization of the overall changes in metabolism, patients were differentiated by DAS-28 score. The DAS-28 score is a composite measure of disease activity within RA, often considered to be a reference standard in patient assessment that reflects swelling and tenderness in the 28 joints measured in this assessment, along with ESR and the patient’s global health assessment [11]. Patients were first stratified by an overall reduction in disease score of 1.2, reflecting a clinically significant improvement in disease activity associated with initiation of MTX. This data was further corroborated by meeting the DAS-28 score of less than 2.6, the threshold clinically recognized as remission [12,13]. Stratification of patients according to a change of −1.2 in their DAS-28 score resulted in two groups deemed responders (*n* = 11) and non-responders (*n* = 9). This knowledge was then applied to the overall metabolic changes in order to identify key metabolites associated with response to MTX therapy and the pharmacological effects of MTX.

Prior to identification of metabolites associated with the pharmacological effects of MTX, we must first address the overall metabolic changes observed in RA and following initiation of MTX therapy. Significant differences were observed in the plasma metabolome of RA patients as compared with healthy control subjects to RA, many of which were associated with fatty acid/lipid metabolism. This is not surprising, as dyslipidemia is a common feature among a variety of rheumatic diseases, and lipids have been shown to play an important role in adaptive immunity and inflammation [14,15,16]. RA is known to promote a global inflammatory state, stimulating lipolysis and reducing lipid accumulation leading to insulin resistance (IR) [17]. This was observed in our dataset, with decreases in both saturated and unsaturated triglycerides occurring in active RA and a corresponding increase in fatty acids. While one group has reported increased levels of triglycerides in RA [18], most investigators have reported that triglycerides were reduced in RA as compared with healthy control subjects [19,20,21]. Following MTX therapy, a correction towards the healthy control levels of triglycerides were observed in our study. Among the 35 metabolites of interest identified in volcano plots to show a correction towards the healthy control following MTX therapy (*p* < 0.05), 24 (69%) metabolites were unsaturated and saturated triglycerides. Hence, a strong correlation was found between a decrease in DAS-28 score and increasing levels of triglycerides. According to the Spearman’s correlation analysis (Table S5), 29 triglyceride metabolites, accounting for 71% of significant metabolites identified, inversely correlate with disease activity based on the DAS-28 score.

Consistent with increased lipolysis in RA, increases in both saturated and unsaturated fatty acids were observed in RA as compared with healthy control subjects. Elevated levels of fatty acids in RA have also been previously observed [8]. Initiation of MTX therapy was found to result in significant decreases in saturated and unsaturated fatty acids, with a return towards levels observed in the healthy control subjects. The oxidation of fatty acids and their metabolites have been implicated in vascular damage in autoimmune disorders such as RA [22]. Previous observations of decreases in fatty acids following MTX therapy, along with a corresponding reduction in systemic inflammation, supported the Cardiovascular Inflammation Reduction Trial (CIRT) study that evaluated the cardioprotective effects of MTX [23]. While it was found that low dose MTX was not associated with fewer cardiovascular events in the study population, it may have been due to a lack of inflammation and lack of dysregulation of fatty acid/lipid metabolism within the baseline population for the study. The correction observed in fatty acid/lipid metabolism likely contributes to insulin sensitivity and the cardioprotective profile of MTX.

In addition to triglycerides, further dysregulation of fatty acid/lipid metabolism was observed in RA and in response to MTX therapy. Levels of lysophosphatidylcholines (LPC) were found to be higher in patients with RA, consistent with previous observations [21]. High levels of LPC in plasma have been shown to be a reliable measure of inflammation [24]. Following MTX therapy, decreases in LPC were observed, correcting towards the healthy control levels. Acylcarnitine, a metabolite necessary in lipid metabolism responsible for the transport of long-chain fatty acids, was found to be increased in association with RA, and has been previously reported [25,26]. Initiation of MTX therapy resulted in a reduction of acylcarnitine levels. Taken together, the data support a marked disruption of fatty acid/lipid metabolism that occurs during active disease and provide evidence of a shift away from glucose metabolism known to be associated with RA [26]. The normalization of fatty acid/lipid metabolism following initiation of MTX towards levels observed in a healthy control population, may indicate a correction in the dysregulation of glucose metabolism associated with pharmacological response to MTX therapy.

In addition to the changes of metabolites involved in fatty acid/lipid metabolism, levels of amino acids, phosphates, and other organic acid classes have been shown to be increased in the context of RA [25]. Specifically, levels of α-ketoglutarate have been shown to increase in RA [27,28], which was also observed in our dataset. Levels of α-ketoglutarate have been shown to accumulate under hypoxic conditions, which may play a role in chronic inflammation, a characteristic of RA. A modest reduction of levels of α-ketoglutarate were observed following MTX therapy, however, it was not found to be statistically significant. In addition to observations consistent with previous reports, a subset of our data conflicts with what has been previously reported, specifically, statistically significant decreases were observed only in threonine (*p* = 0.0024) and arginine (*p* = 1.12 × 10^−5^), in patients with RA, which is inconsistent with increased levels of tryptophan [8,20], threonine, aspartic acid, glycine, and arginine that have been reported previously [29]. The observed decreases in amino acids in our study may be explained by the fact that highly metabolic tissues consume more nutrients due to the increased metabolism of activated cells, such as proinflammatory metabolites, or to resolve inflammation via anti-inflammatory metabolites, inevitably resulting in a decrease in circulating metabolites [30]. Arginine is also involved in two metabolic pathways critical to RA disease pathogenesis, i.e., nitric oxide synthase uncoupling and citrullination. The conversion of arginine to citrulline has been shown to be upregulated in synovial fluid of patients with RA due to a process known as” hypercitrullination” [31]. The observed reduction of arginine in RA may be due to both hypercitrullination, and arginine’s role in the production of reactive oxygen species (ROS), a hallmark trait of inflamed tissues [32,33,34]. A modest increase in both arginine and threonine levels occurred following initiation of MTX therapy, though neither was found to be statistically significant.

After identifying changes in the plasma metabolome associated with RA and MTX therapy, we turned our attention towards metabolic changes associated with response to MTX therapy, to determine key pathways involved in the pharmacological response to MTX. This was accomplished by first differentiating responders (*n* = 11) and non-responders (*n* = 9) according to a change of −1.2 in their DAS-28 score. Then, the log normalized change in peak intensity of all metabolites from baseline to 16 weeks post MTX therapy were stratified according to responders and non-responders. This led to identification of 19 statistically significant metabolites (*p* < 0.05) associated with MTX therapy that differentiated responders from non-responders. In addition to the previously identified dysregulation of fatty acid/lipid metabolism, metabolites found to be associated with response to MTX fell into two additional categories, i.e., nucleotide metabolism and energy metabolism, which are both metabolic pathways previously shown to be associated with MTX’s projected mechanism of action [14,35].

The normalization of fatty acid metabolism and its association with the pharmacological response to MTX therapy is clearly indicated by the number of metabolites shown to differentiate responders from non-responders. Those statistically significant metabolites (*p* < 0.05) involved in fatty acid/lipid metabolism shown to differentiate responders and non-responders according to δDAS-28 (−1.2) include glucosylceramide (GlcCer, *n* = 3), phosphadtidylcholines (PC, *n* = 5), sphingomyelins (SM, *n* = 5), and phosphatidylethanolamines (PE, *n* = 2). When cross referenced with those metabolites found to differentiate patients in clinical remission from those not in clinical remission, i.e., responders according to the DAS-28 threshold of 2.6, the most significant discriminating metabolites include increased levels of choline (*p* = 0.0076) and decreases in levels of multiple glucosylceramides (*p* = 0.0175, 0.0066, and 0.0037). Evidence of the role of choline and glucosylceramide in the pharmacological response to MTX, and as a potential therapeutic biomarker of MTX response, is further strengthened by the ROC curve analysis, with choline (AUC = 0.77) and GlcCer d41:1 (AUC = 0.71) showing a marked separation in responders and non-responders following 16 weeks of MTX therapy.

There has been increasing evidence of the role of the cholinergic system and its role in RA [33], with studies currently underway on the role of choline as a potential diagnostic or prognostic biomarker of RA [36]. Activation of the cholinergic receptor inhibits the synthesis of proinflammatory cytokines but does not inhibit the synthesis of anti-inflammatory cytokines [37]. Increased levels of choline observed in responders to MTX therapy (*p* = 0.0076) may play a role in the activation of anti-inflammatory cytokines and subsequent reduction in disease activity score. In addition, choline is required for the biosynthesis of phosphatidylcholine, a necessary component in cell membranes, which has been shown to decrease arthritis in established disease [25]. Choline is also known to play a role in one-carbon metabolism and is responsible for methylation of homocysteine to methionine [38]. In our study, levels of methionine were found to decrease in active RA relative to the healthy control subjects (*p* = 7.4 × 10^−7^), however, they were not observed to increase following 16 weeks of MTX therapy. One-carbon metabolism is necessary for DNA synthesis, a process known to be dysregulated due to MTX therapy, and perturbations of one-carbon metabolism has been shown to affect inflammatory processes [39,40]. The higher levels of choline in responders makes a strong case for choline’s role in the therapeutic response to methotrexate.

Nucleic acid metabolism is also believed to be related to the therapeutic effects of MTX. MTX acts as a folate antagonist, inhibiting dihydrofolate reductase (DHFR), setting off a cascade of reactions, leading to the inhibition of de novo purine and pyrimidine synthesis [14,41,42,43]. Multiple metabolites involved in nucleotide metabolism were shown to differentiate MTX responders from non-responders including hypoxanthine, inosine, and guanosine. It is worthwhile noting the interrelated nature of these metabolites, both guanosine and inosine serve as purine nucleotides, and inosine is formed through phosphorylation of hypoxanthine. Previous reports have indicated an increase in hypoxanthine in RA relative to a healthy control group [43]. While an increase in levels of hypoxanthine was observed in RA, the differences observed were not statistically significant (*p* = 0.75). The lack of statistical significance may be due to the limited size of the cohort for this study. In fact, no significant differences were observed in hypoxanthine, inosine, or guanosine in RA patients as compared with healthy control subjects. In addition, these metabolites were not found to be significantly altered following initiation of MTX therapy. Despite this, all three metabolites displayed increased levels in responders as compared with non-responders (Figure 6), with all three metabolites having an AUC > 0.70 according to the ROC curve analysis (Table S7). Among the metabolites found to differentiate responders and non-responders, hypoxanthine was the only metabolite also identified by Spearman’s correlation analysis to display an inverse correlation with disease activity (*p* = 0.036, Table S5). The overall effects on hypoxanthine, guanosine, and inosine may suggest downstream effects on purine and pyrimidine rations, resulting in dysregulation of cell metabolism, energy conservation and biosynthetic pathways, as well as signal transductions and translation. In addition, adenosine is known to mediate effects on inflammatory cells, with adenosine receptors changing in active RA [44]. Once formed or released into the extracellular space, adenosine can be deaminated to inosine, a potential explanation for the increased levels of inosine and guanosine observed in responders to MTX therapy. The elevated levels of all three metabolites observed in the plasma of responders may also be associated with a reduction in oxidative stress, a hallmark trait of RA [44,45]. One prevailing theory of the mechanism by which MTX exerts its effects in RA is through the inhibition of 5-aminoimidazole-4-carboxamide ribonucleotide (AICAR) transformylase (ATIC), leading to the release of adenosine nucleotides, and increased levels of adenosine, which display potent inhibitory effects on inflammatory cells [14]. The elevated levels of inosine and hypoxanthine observed in responders to MTX therapy may provide support for this hypothesized mechanism.

Metabolites involved in energy metabolism shown to differentiate responders and non-responders that are statistically significant (*p* < 0.05) include itaconic acid (IA) and nicotinamide. IA is a metabolite produced from the tricarboxylic acid (TCA) cycle and is an intermediate of cis-aconitic acid. IA has been shown to inhibit succinate dehydrogenase, thereby, inducing metabolic rewiring during proinflammatory activation [35]. In our study, we found decreased levels of IA in those that responded to MTX therapy (*p* = 0.026) following 16 weeks of MTX therapy. A strong correlation between decreasing levels of IA and decreased disease activity has been previously reported [46]. These data are also corroborated by a previous report that indicates high levels of IA may serve as a potential biomarker of early RA [47]. Nicotinamide has been shown to have implications in inflammation, and RA and is involved in multiple metabolic processes known to be dysregulated by MTX therapy, including glycolysis, TCA, and the electron transport chain (ETC) [48,49]. Changes in metabolite levels of nicotinamide were not found to be statistically significant (*p* > 0.25) when comparing RA patients to the healthy cohort or following MTX therapy. However, changes in nicotinamide following the initiation of MTX were shown to differentiate responders from non-responders, displaying a marked increase in responders. The higher levels of nicotinamide observed in responders may be due to a correction in the dysregulation of glycolysis, TCA, and ETC in response to MTX therapy.

## 4. Materials and Methods

### 4.1. Patients

Biobanked, plasma samples were acquired from a subset of RA patients (*n* = 20) that participated in a 16-week open-label study that sought to identify predictors of MTX response in RA [50]. The study included sites within the Rheumatology Arthritis Investigational Network (RAIN). For each patient, plasma samples at baseline and 16 weeks were provided for analysis. All patients received 15 mg/week of MTX and 1 mg/day of folic acid upon enrollment. The MTX dose was escalated to 20 mg/week in patients that did not achieve clinical remission by 8 weeks, as tolerated. Eligibility for participation included: ≥19 years of age, fulfillment of the 1987 American College of Rheumatology criteria for RA, no prior exposure to MTX within the last 12 months, no or stable doses of prednisone for the last 2 weeks (not to exceed 10 mg/day of prednisone), and receiving no or stable doses of non-steroidal anti-inflammatory drugs for the last week [51]. Patients excluded from the study included: patients with other rheumatic diseases (i.e., Sjogren’s, SLE, and overlap connective tissue disease), pregnant or lactating women, women of childbearing age not practicing an effective method of contraception, patients with a history of alcohol abuse who are unwilling to abstain or limit alcohol consumption, and patients with cytopenias or abnormal hepatic or renal function at the time of screening. Healthy control plasma samples were collected from volunteers reporting well to excellent health [52]. All patients provided informed consent and the studies were conducted under the University of Nebraska Medical Center Institutional Review Board approved protocols.

### 4.2. Clinical Data

Clinical data were collected on study participants at both baseline and at Week 16, at the follow-up visits, and included the patient’s global health assessment (0–100 mm visual analog scale (VAS0), the 28-joint swollen and tender joint counts (SJC and TJC), the erythrocyte sedimentation rate (ESR, mm/hr), and the DAS-28 ESR composite disease activity score [53]. The primary response outcome for this study was defined as an improvement in DAS-28 (<−1.2 units) and achievement of remission (DAS-28 < 2.6) was used an additional measure of response [13,54].

### 4.3. Metabolomics Analysis

Plasma samples were submitted for semi-targeted metabolomic analysis to the National Institutes of Health (NIH) West Coast Metabolomics Center at the University of California, Davis (Davis, CA). Plasma was analyzed using three independent analytical methods for the relative quantification of intermediates of primary metabolism, biogenic amines, and lipids [55,56]. Samples were prepared for analysis using a biphasic liquid-liquid extraction protocol standardized for global metabolomic profiling in plasma and serum samples across the three analytical platforms used in this study. Intermediates of primary metabolism were measured by automated linear exchange-cold injection system gas chromatography time-of-flight mass spectrometry. Biogenic amines were analyzed by hydrophilic interaction liquid chromatography electrospray ionization quadrupole time-of-flight mass spectrometry. Lipids were analyzed by charged surface hybrid chromatography electrospray ionization quadrupole time-of-flight mass spectrometry. Peak identification was based on retention time and mass spectral data from MassBank of North America [56]. Peak height intensity tables were curated by the NIH West Coast Metabolomics Center and submitted to Metabolomics Workbench (https://www.metabolomicsworkbench.org/, access on 20 October 2021) under Project ID 2895. The resulting raw peak intensity data were obtained, and a standardized normalization procedure was subsequently performed. The normalization ratio was calculated as the ratio of the sum of all peak heights for identified metabolites (mTIC) to the average mTIC for all samples. Then, the observed peak heights for each metabolite were divided by the normalization ratio, providing the normalized peak height intensity. Duplicate metabolites (those observed in more than one analytical platform) were combined by mean normalization to ensure equal weighting to a given platform, and then averaged. The resulting normalized peak height intensities were uploaded into MetaboAnalyst (Version 5.0). Then, the data were normalized by logarithmic transformation and auto-scaled according to unit variance [57,58], analyzed for fold change, and visualized via principal components analysis (PCA), partial least squares discriminant analysis (PLS-DA), and volcano plots in order to identify and differentiate those metabolites altered in active RA and with MTX therapy.

### 4.4. Enrichment Analysis

The fold change values and *p*-values obtained from MetaboAnalyst were used to analyze identified metabolites via chemical and metabolic network analysis. Visualization of chemometric and biochemical network maps associated with induction of RA and MTX therapy were conducted using MetaMapp 2020. The network maps were generated based upon chemical similarity utilizing the Kyoto Encyclopedia of Genes and Genomes (KEGG) metabolic network database and Tanimoto substructure similarity coefficients [57]. Then, the processed data were visualized in Cytoscape (Version 3.7.2, Institute for Systems Biology, Seattle, Washington, DC, USA) Further enrichment analysis was performed utilizing the open-source software “Chemical Similarity Enrichment Analysis for Metabolomics” or “ChemRICH”. ChemRICH (University of California, Davis; Davis, CA, USA) utilizes chemical ontologies and structural similarities to generate nonoverlapping sets of identified metabolites. This method does not rely upon the size of a background database or defined biochemical pathways [58].

### 4.5. Statistical Analysis

The analyses of identified metabolites were evaluated by both univariate and multivariate analyses utilizing MetaboAnalyst (Version 5.0, McGill University; Montreal, Quebec, Canada). Metabolites were evaluated for fold change and statistical significance. A threshold of significance was set at a *p*-value of less than 0.05 and was used in both metabolomic and chemometric metabolic enrichment analyses. The chemometric analysis was accomplished using ChemRICH, with metabolites of significant interest having a *p*-value of <0.05. Statistical analyses, including linear regression and Spearman’s rank correlation, were performed utilizing JMP Pro 15 (SAS Institute, Cary, NC, USA).

## 5. Conclusions

The data obtained from this study were intended for the generation of hypothesis regarding the effects on the plasma metabolome involved in RA progression and in response to MTX therapy. It is necessary to note that while as many variables as possible were controlled for, metabolomic analysis has been shown to be sensitive to diet and physical activity [59]. The power of this study is restricted due to the relatively small sample size, and further conclusions must be drawn from a larger and more diverse cohort. Despite this, significant changes in the plasma metabolome were observed in association with RA, and in response to MTX therapy. Many important changes in a variety of metabolic pathways occur in RA, with a return towards normalized levels observed following MTX therapy, especially metabolites involved in fatty acid/lipid metabolism, nucleic acid metabolism, and energy metabolism. Key metabolites involved in purine and pyrimidine biosynthesis and the citric acid cycle appear to be critical in the pharmacological response to MTX. Future work will focus on identification of the roles of hypoxanthine and nicotinamide in MTX therapy, and determining if additional metabolites may serve as suitable clinical biomarkers for the prediction of MTX efficacy in RA.

## Figures and Tables

**Table 1:** Demographic data for study population. RA patients were characterized as “responders” and “non-responders” based on a reduction in DAS-28 of greater than 1.2 (δDAS-28 (<−1.2)). Data are presented as median [IQR] unless otherwise noted. Swollen joint count (SJC); tender joint count (TJC); erythrocyte sedimentation rate (ESR); disease activity score (DAS-28).

**Study Cohort**	**Control**	**RA Patients**	***p*-Value**
Subjects, *n*	20	20	―
Female, *n* (%)	17 (85%)	14 (70%)	0.77
Age (years)	49 [44, 57]	52 [41, 65]	0.99
Current smoker, *n* (%)	1 (5%)	4 (20%)	0.31
Symptoms to diagnosis (months)	―	11 [5, 10]	―
Diagnosis to MTX (months)	―	2 [0, 0]	―
SJC (0–28)	―	5 [1, 7]	―
TJC (0–28)	―	6 [0, 7]	―
ESR (mm/h)	―	25 [12, 32]	―
Global health assessment (0–100 mm)	―	39 [19, 56]	―
DAS-28	―	4.1 [3.0, 5.4]	―
**RA–Baseline**	**Non-Responders**	**Responders**	***p*-Value**
RA patients, *n*	9	11	―
Female, *n* (%)	8 (73%)	7 (70%)	0.77
Age (years)	57 [45, 72]	48 [27, 63]	0.3
Current smoker, *n* (%)	3 (33%)	1 (9%)	0.06
SJC	6 [3, 7]	10 [6, 14]	0.3
TJC	6 [2, 7]	11 [6, 17]	0.08
ESR (mm/hr)	23 [14, 24]	31 [13, 43]	0.4
Global health assessment	50 [38, 52]	48 [34, 63]	0.8
DAS-28	4.6 [4.1, 5.5]	5.5 [4.8, 6.2]	0.08
**RA–Week 16**	**Non-Responders**	**Responders**	***p*-Value**
SJC	4 [1, 5]	0 [0, 1]	0.05
TJC	6 [1, 5]	1 [0, 0]	0.01
Global health assessment	48 [39, 57]	15 [5, 14]	0.0003
ESR (mm/hr)	31 [17, 33]	15 [8, 19]	0.1
DAS-28	4.3 [3.7, 5.1]	2.2 [1.6, 2.5]	0.001
Remission (DAS-28 < 2.6), *n* (%)	0 (0%)	8 (73%)	0.0002

## Data Availability

The datasets generated and analyzed for this study can be found in the Metabolomics Workbench database (https://www.metabolomicsworkbench.org/, accessed on 20 October 2021) under Project ID 2895.

## Supplementary Materials

The following are available online at https://www.mdpi.com/article/10.3390/metabo11120824/s1, Table S1: Statistically significant metabolites (*p* < 0.05) associated with induction of RA, Table S2: Statistically significant metabolites (*p* < 0.055) associated with MTX therapy, Table S3: Chemometric enrichment analysis of metabolites associated with induction of RA, Table S4: Chemometric enrichment analysis of metabolites associated MTX therapy, Table S5: Metabolite levels displaying a statistically significant inverse correlation (*p* < 0.05) with DAS-28, Table S6: Metabolites of interest (*p* < 0.05) identified in volcano plots to display a correction towards healthy control following MTX therapy, Table S7: ROC analysis of metabolite levels between responders and non-responders defined by δDAS-28 (−1.2) after 16 weeks of MTX therapy.

## References

[1] Lee D.M., Weinblatt M.E. (2001). Rheumatoid arthritis. Lancet.

[2] Castro-Santos P., Laborde C.M., Díaz-Peña R. (2015). Genomics, proteomics and metabolomics: Their emerging roles in the discovery and validation of rheumatoid arthritis biomarkers. Clin. Exp. Rheumatol..

[3] Cross M., Smith E., Hoy D., Carmona L., Wolfe F., Vos T., Williams B., Gabriel S., Lassere M., Johns N. (2014). The global burden of rheumatoid arthritis: Estimates from the global burden of disease 2010 study. Ann. Rheum. Dis..

[4] Fraenkel L., Bathon J.M., England B.R., St Clair E.W., Arayssi T., Carandang K., Deane K.D., Genovese M., Huston K.K., Kerr G. (2021). 2021 American College of Rheumatology Guideline for the Treatment of Rheumatoid Arthritis. Arthritis Rheumatol..

[5] Moreland L.W., O’Dell J.R., Paulus H.E., Curtis J.R., Bathon J.M., St Clair E.W., Bridges S.L., Zhang J., McVie T., Howard G. (2012). A randomized comparative effectiveness study of oral triple therapy versus etanercept plus methotrexate in early aggressive rheumatoid arthritis: The treatment of Early Aggressive Rheumatoid Arthritis Trial. Arthritis Rheum..

[6] Funk R.S., Becker M.L. (2016). Disease modifying anti-rheumatic drugs in juvenile idiopathic arthritis: Striving for individualized therapy. Expert Rev. Precis. Med. Drug Dev..

[7] Guma M., Tiziani S., Firestein G.S. (2016). Metabolomics in rheumatic diseases: Desperately seeking biomarkers. Nat. Rev. Rheumatol..

[8] Zhou J., Chen J., Hu C., Xie Z., Li H., Wei S., Wang D., Wen C., Xu G. (2016). Exploration of the serum metabolite signature in patients with rheumatoid arthritis using gas chromatography-mass spectrometry. J. Pharm. Biomed. Anal..

[9] Rinschen M.M., Ivanisevic J., Giera M., Siuzdak G. (2019). Identification of bioactive metabolites using activity metabolomics. Nat. Rev. Mol. Cell Biol..

[10] Funk R.S., Singh R.K., Becker M.L. (2020). Metabolomic Profiling to Identify Molecular Biomarkers of Cellular Response to Methotrexate In Vitro. Clin. Transl. Sci..

[11] England B.R., Tiong B.K., Bergman M.J., Curtis J.R., Kazi S., Mikuls T.R., O’Dell J.R., Ranganath V.K., Limanni A., Suter L.G. (2019). 2019 Update of the American College of Rheumatology Recommended Rheumatoid Arthritis Disease Activity Measures. Arthritis Care Res. (Hoboken).

[12] Van Riel P.L. (2014). The development of the disease activity score (DAS) and the disease activity score using 28 joint counts (DAS-28). Clin. Exp. Rheumatol..

[13] Prevoo M.L., Van’T Hof M.A., Kuper H.H., van Leeuwen M.A., van de Putte L.B., van Riel P.L. (1995). Modified disease activity scores that include twenty-eight-joint counts. Development and validation in a prospective longitudinal study of patients with rheumatoid arthritis. Arthritis Rheum..

[14] Cronstein B.N., Aune T.M. (2020). Methotrexate and its mechanisms of action in inflammatory arthritis. Nat. Rev. Rheumatol..

[15] O’Neill L.A.J., Kishton R.J., Rathmell J. (2016). A guide to immunometabolism for immunologists. Nat. Rev. Immunol..

[16] Nomura M., Liu J., Rovira I.I., Gonzalez-Hurtado E., Lee J., Wolfgang M.J., Finkel T. (2016). Fatty acid oxidation in macrophage polarization. Nat. Immunol..

[17] Arias de la Rosa I., Escudero-Contreras A., Rodríguez-Cuenca S., Ruiz-Ponce M., Jiménez-Gómez Y., Ruiz-Limón P., Pérez-Sánchez C., Ábalos-Aguilera M.C., Cecchi I., Ortega R. (2018). Defective glucose and lipid metabolism in rheumatoid arthritis is determined by chronic inflammation in metabolic tissues. J. Intern. Med..

[18] Situnayake R.D., Kitas G. (1997). Dyslipidaemia and Rheumatoid Arthritis. Ann. Rheum. Dis..

[19] Svenson K.L., Lithell H., Hallgren R., Selinus I., Vessby B. (1987). Serum lipoprotein in active rheumatoid arthritis and other chronic inflammatory arthritides. I Relativity to inflammatory activity. Arch. Intern. Med..

[20] Svenson K.L., Lithell H., Hallgren R., Selinus I., Vessby B. (1987). Serum lipoprotein in active rheumatoid arthritis and other chronic inflammatory arthritides. II. Effects of anti-inflammatory and disease-modifying drug treatment. Arch. Intern. Med..

[21] Rantapaa-Dahlqvist S., Wallberg-Jonsson S., Dahlen G. (1991). Lipoprotein (a), lipids and lipoproteins in patients with rheumatoid arthritis. Ann. Rheum. Dis..

[22] Chen W., Wang Q., Zhou B., Zhang L., Zhu H. (2021). Lipid Metabolism Profiles in Rheumatic Diseases. Front. Pharmacol..

[23] Ridker P.M., Everett B.M., Pradhan A., MacFadyen J.G., Solomon D.H., Zaharris E., Mam V., Hasan A., Rosenberg Y., Iturriaga E. (2019). Low-Dose Methotrexate for the Prevention of Atherosclerotic Events. N. Engl. J. Med..

[24] Knuplez E., Marsche G. (2020). An Updated Review of Pro- and Anti-Inflammatory Properties of Plasma Lysophosphatidylcholines in the Vascular System. Int. J. Mol. Sci..

[25] Li J., Che N., Xu L., Zhang Q., Wang Q., Tan W., Zhang M. (2018). LC-MS-based serum metabolomics reveals a distinctive signature in patients with rheumatoid arthritis. Clin. Rheumatol..

[26] Lazarevic M.B., Vitic J., Mladenovic V., Myones B.L., Skoset J.L., Swedler W.I. (1992). Dyslipoproteinemia in the course of active rheumatoid arthritis. Semin. Arthritis Rheum..

[27] Falconer J., Murphy A.N., Young S.P., Clark A.R., Tiziani S., Guma M., Buckley C.D. (2018). Review: Synovial Cell Metabolism and Chronic Inflammation in Rheumatoid Arthritis. Arthritis Rheumatol..

[28] Wang M., Huang J., Fan H., He D., Zhao S., Shu Y., Li H., Liu L., Lu S., Xiao C. (2018). Treatment of rheumatoid arthritis using combination of methotrexate and Tripterygium glycosides tablets—A quantitative plasma pharmacochemical and pseudo targeted metabolomic approach. Front. Pharmacol..

[29] Mills E., O’Neill L.A. (2014). Succinate: A metabolic signal in inflammation. Trends Cell Biol..

[30] Li Y., Zheng J.Y., Liu J.Q., Yang J., Liu Y., Wang C., Ma X., Liu B., Xin G., Liu L. (2016). Succinate/NLPR3 Inflammasome Induces Synovial Fibroblast Activation: Therapeutical Effects of Clematichinenoside AR on Arthritis. Front. Immunol..

[31] Gazitt T., Lood C., Elkon K.B. (2016). Citrullination in Rheumatoid Arthritis-A Process Promoted by Neutrophil Lysis?. Rambam Maimonides Med. J..

[32] Smolenska Z., Smolenski R.T., Zdrojewski Z. (2016). Plasma concentrations of amino acid and nicotinamide metabolites in rheumatoid arthritis--potential biomarkers of disease activity and drug treatment. Biomarkers.

[33] Coras R., Murillo-Saich J.D., Guma M. (2020). Circulating Pro- and Anti-Inflammatory Metabolites and Its Potential Role in Rheumatoid Arthritis Pathogenesis. Cells.

[34] Chalupsky K., Cai H. (2005). Endothelial dihydrofolate reductase: Critical for nitric oxide bioavailability and role in angiotensin II uncoupling of endothelial nitric oxide synthase. Proc. Natl. Acad. Sci. USA.

[35] Crabtree M.J., Tatham A.L., Hale A.B., Alp N.J., Channon K.M. (2009). Critical role for tetrahydrobiopterin recycling by dihydrofolate reductase in regulation of endothelial nitric-oxide synthase coupling: Relative importance of the de novo biopterin synthesis versus salvage pathways. J. Biol. Chem..

[36] Guma M. (2015). Choline Metabolites as Biomarkers in Rheumatoid Arthritis.

[37] Pan X.H., Zhang J., Yu X., Qin L., Kang L., Zhang P. (2010). New therapeutic approaches for the treatment of rheumatoid arthritis may rise from the cholinergic anti-inflammatory pathway and antinociceptive pathway. Sci. World J..

[38] Borovikova L.V., Ivanova S., Zhang M., Yang H., Botchkina G.I., Watkins L.R., Wang H., Abumrad N., Eaton J.W., Tracey K.J. (2000). Vagus nerve stimulation attenuates the systemic inflammatory response to endotoxin. Nature.

[39] Emmerson J.T., Murray L.K., Jadavji N.M. (2017). Impact of dietary supplementation of one-carbon metabolism on neural recovery. Neural Regen. Res..

[40] Abbenhardt C., Miller J.W., Song X., Brown E.C., Cheng T.-Y.D., Wener M.H., Zheng Y., Toriola A., Neuhouser M.L., Beresford S.A.A. (2013). Biomarkers of one-carbon metabolism are associated with biomarkers of inflammation in women. J. Nutr..

[41] Tian H., Cronstein B.N. (2007). Understanding the mechanisms of action of methotrexate: Implications for the treatment of rheumatoid arthritis. Bull. NYU Hosp. Jt. Dis..

[42] Taflin H., Wettergren Y., Odin E., Derwinger K. (2014). Folate levels measured by LC-MS/MS in patients with colorectal cancer treated with different leucovorin dosages. Cancer Chemother Pharm..

[43] Yuan L., Sharer J.D. (2016). Quantitative Analysis of Total Plasma Homocysteine by LC-MS/MS. Curr. Protoc. Hum. Genet..

[44] Madsen R.K., Lundstedt T., Gabrielsson J., Sennbro C.-J., Alenius G.-M., Moritz T., Rantapää-Dahlqvist S., Trygg J. (2011). Diagnostic properties of metabolic perturbations in rheumatoid arthritis. Arthritis Res..

[45] Cronstein B.N., Sitkovsky M. (2017). Adenosine and adenosine receptors in the pathogenesis and treatment of rheumatic diseases. Nat. Rev. Rheumatol..

[46] Daly R., Blackburn G., Best C., Goodyear C.S., Mudaliar M., Burgess K., Stirling A., Porter D., McInnes I.B., Barrett M.P. (2020). Changes in Plasma Itaconate Elevation in Early Rheumatoid Arthritis Patients Elucidates Disease Activity Associated Macrophage Activation. Metabolites.

[47] Michopoulos F., Karagianni N., Whalley N.M., Firth M.A., Nikolaou C., Wilson I., Critchlow S.E., Kollias G., Theodoridis G.A. (2016). Targeted Metabolic Profiling of the Tg197 Mouse Model Reveals Itaconic Acid as a Marker of Rheumatoid Arthritis. J. Proteome Res..

[48] Wang Z., Chen Z., Yang S., Wang Y., Yu L., Zhang B., Rao Z., Gao J., Tu S. (2012). (1)H NMR-based metabolomic analysis for identifying serum biomarkersto evaluate methotrexate treatment in patients with early rheumatoid arthritis. Exp. Ther. Med..

[49] Meiser J., Kraemer L., Jaeger C., Madry H., Link A., Lepper P.M., Hiller K., Schneider J.G. (2018). Itaconic acid indicates cellular but not systemic immune system activation. Oncotarget.

[50] O’Dell J.R. Treatment of Rheumatoid Arthritis with Methotrexate: Predictors of Response.

[51] Arnett F.C., Edworthy S.M., Bloch D.A., McShane D.J., Fries J.F., Cooper N.S., Healey L.A., Kaplan S.R., Liang M.H., Luthra H.S. (1988). The American Rheumatism Association 1987 revised criteria for the classification of rheumatoid arthritis. Arthritis Rheum..

[52] Luedders B.A., Johnson T.M., Sayles H., Thiele G.M., Mikuls T.R., O’Dell J.R., England B.R. (2020). Predictive ability, validity, and responsiveness of the multi-biomarker disease activity score in patients with rheumatoid arthritis initiating methotrexate. Semin. Arthritis Rheum..

[53] Anderson D.R., Duryee M.J., Shurmur S.W., Um J.Y., Bussey W.D., Hunter C.D., Garvin R.P., Sayles H.R., Mikuls T.R., Klassen L.W. (2014). Unique antibody responses to malondialdehyde-acetaldehyde (MAA)-protein adducts predict coronary artery disease. PLoS ONE.

[54] Van Gestel A.M., Prevoo M.L., Van’T Hof M.A., Van Rijswijk M.H., Van de Putte L.B., Van Riel P.L. (1996). Development and validation of the European League Against Rheumatism response criteria for rheumatoid arthritis. Comparison with the preliminary American College of Rheumatology and the World Health Organization/International League against Rheumatism Criteria. Arthritis Rheum..

[55] Fiehn O. (2016). Metabolomics by Gas Chromatography-Mass Spectrometry: Combined Targeted and Untargeted Profiling. Curr. Protoc. Mol. Biol..

[56] Cajka T., Fiehn O. (2016). Toward Merging Untargeted and Targeted Methods in Mass Spectrometry-Based Metabolomics and Lipidomics. Anal. Chem..

[57] Blaženović I., Kind T., Ji J., Fiehn O. (2018). Software Tools and Approaches for Compound Identification of LC-MS/MS Data in Metabolomics. Metabolites.

[58] Barupal D.K., Fiehn O. (2017). Chemical Similarity Enrichment Analysis (ChemRICH) as alternative to biochemical pathway mapping for metabolomic datasets. Sci. Rep..

[59] Scalbert A., Brennan L., Fiehn O., Hankemeier T., Kristal B.S., van Ommen B., Pujos-Guillot E., Verheij E., Wishart D., Wopereis S. (2009). Mass-spectrometry-based metabolomics: Limitations and recommendations for future progress with particular focus on nutrition research. Metab. Off. J. Metab. Soc..

