# Supplementary material for: Plasma Metabolome Normalization in Rheumatoid Arthritis Following Initiation of Methotrexate and the Identification of Metabolic Biomarkers of Efficacy
Source: Metabolites. 2021 Nov 30;11(12):824. doi: 10.3390/metabo11120824 (PMC8706490; doi:10.3390/metabo11120824)
Supplement: Supplementary file 1 [file metabolites-11-00824-s001.zip › metabolites-1469504-supplementary.pdf]

Supplementary Materials:

Table S1. Metabolites associated with induction of RA. Lysophosphatidylcholines (LPC); carnitines (CAR); fatty acid (FA), phosphatidylinositol (PI); ceramides (Cer); phosphatidylethanolamines (PE-P, PE); triglycerides (TG); sphingomyelins (SM); phosphatidylcholine (PC); glucosyl ceramide (GlcCer).

Control V. Disease

Compound	Class	FC	q value	p value
Orthanilic Acid	Sulfanilic Acids	2.901	3.33E-06	3.09E-09
Homogentisic Acid	Carbocyclic Acids	0.29183	3.33E-06	1.01E-08
Glyceric Acid	Sugar Acids	1.7908	5.50E-06	2.52E-08
Succinic Acid	Succinates	2.6532	1.06E-05	6.08E-08
2-Hydroxyglutaric Acid	Glutarates	2.6508	2.71E-05	2.09E-07
Methionine	Sulfur Amino Acids	0.57353	7.97E-05	6.71E-07
Malic Acid	Dicarboxylic Acids	2.0396	8.70E-05	9.33E-07
Erythronolactone	Butyrates	2.1319	0.00010942	1.31E-06
CAR 18:1	Carnitine	4.2412	0.00022897	1.74E-06
FA 22:2	Unsaturated Fatty Acids	2.1546	0.00023179	3.57E-06
Maltose	Disaccharides	1.6945	0.00024359	8.72E-07
2,5-Dihydroxypyrazine	Pyridine	1.55	0.00024359	4.14E-06
Gluconic Acid	Gluconates	1.7402	0.00025468	4.30E-06
CAR 16:0	Carnitine	3.9316	0.00043715	4.87E-06
Fumaric Acid	Dicarboxylic Acids	1.7082	0.00046031	1.06E-05
Arginine	Basic Amino Acids	0.38853	0.00046031	3.34E-06
Glycolic acid	Glycolates	1.5059	0.00053909	9.39E-06
PI 38:5	Phosphatidylinositol	0.53416	0.00056993	1.51E-05
FA 18:3	Unsaturated Fatty Acids	2.9349	0.00057437	1.67E-05
Maltotriitol	Sugar Alcohol	1.7518	0.00057437	1.76E-05
CAR 18:0	Carnitine	2.1031	0.00072055	1.68E-05
Methylacetate	Acetates	2.0544	0.0008323	1.89E-05
Cer d34:1	Unsaturated Ceramides	1.3169	0.0011778	3.64E-05
FA 24:1	Unsaturated Fatty Acids	1.5473	0.00121	3.80E-05
Mannose	Sugar Alcohol	1.895	0.001296	4.88E-05
Cer d36:1	Unsaturated Ceramides	1.5172	0.0013425	4.55E-05
CAR 18:2	Carnitine	4.6587	0.0014412	3.14E-05
8-Oxo-2-Deoxyadenosine	Adenosine	3.0792	0.0014412	6.16E-05
FA 16:1	Unsaturated Fatty Acids	2.7461	0.0015777	6.16E-05
FA 17:1	Unsaturated Fatty Acids	2.3176	0.0017515	6.42E-05
FA 20:1	Unsaturated Fatty Acids	2.1846	0.0017515	7.84E-05
Indole-3-Propionic Acid	Microbiota	0.38781	0.0017515	7.94E-05
2-Monoolein	Diglycerides	1.6204	0.0022803	7.67E-05
6,7-Dihydroxycoumarin	Umbelliferones	1.5988	0.0023616	0.00012244
Alpha-Ketoglutarate	Glutarates	2.0982	0.0027137	0.000133
Trehalose	Sugar Alcohol	1.6382	0.0027761	8.40E-05
2-Deoxyribose	Deoxy Sugars	2.2308	0.0028089	0.00010979
PE P-40:4	Phosphatidylethanolamines	1.6882	0.0028089	0.0001503
Cer d38:1	Unsaturated Ceramides	1.4116	0.0038495	0.00022566

Cer d42:2	Unsaturated Ceramides	1.2926	0.004203	0.0002011
LPC P-18:0	Saturated LPC	1.749	0.0047125	0.00028698
Isothreonic Acid	Carboxylic Acids	1.4529	0.0052189	0.00033168
FA 18:2	Unsaturated Fatty Acids	1.944	0.005456	0.00035482
Phosphate	Polymer	1.6893	0.005456	0.00034795
Tryptophan	Aromatic Amino Acid	0.6129	0.0057061	0.00037443
FA 20:2	Unsaturated Fatty Acids	2.051	0.005883	0.00039887
TG 59:2	Unsaturated Triglycerides	0.56996	0.005883	0.00042158
3,6-Anhydro-D-Galactose	Methylgalactosides	2.6059	0.0093053	0.00044427
FA 14:1	Unsaturated Fatty Acids	2.1018	0.0098197	0.0006105
S-Adenosyl-homocysteine	Adenosine	1.7308	0.0098609	0.00068858
TG 59:3	Unsaturated Triglycerides	0.65035	0.0098609	0.00075036
PE 36:3	Phosphatidylethanolamines	0.6212	0.0098609	0.00070696
Serotonin	Biogenic Monoamines	2.7097	0.009922	0.00045718
PC 34:0	Phosphatidylethanolamines	1.2176	0.010634	0.0008656
Cellobiose	Sugar Alcohol	1.9029	0.010898	0.00086384
Xylulose	Ketoses	0.66478	0.01099	0.00063275
LPC 20:1	Unsaturated LPC	1.6801	0.011215	0.0009049
Indoxyl sulfate	Indoles	0.57382	0.011819	0.0010017
Oleic Acid	Unsaturated Fatty Acids	2.7812	0.011883	0.00093652
2-Deoxytetronic Acid	Carboxylic Acids	1.8825	0.012102	0.00086265
Quinic Acid	Carboxylic Acids	1.7894	0.015127	0.001364
Proline	Imino Acids	0.69317	0.015895	0.0014497
FA 17:0	Saturated Fatty Acids	1.4785	0.016269	0.0013936
CAR 14:2	Carnitine	1.7914	0.016858	0.0016445
Isomaltose	Sugar Acids	0.70709	0.017565	0.0014128
TG 46:4	Unsaturated Triglycerides	0.50559	0.017959	0.0018278
Methylsuccinic Acid	Succinates	1.5209	0.018828	0.0017781
Pyroglutamic Acid	Dipeptides	1.2706	0.018828	0.0019676
Galactonic Acid	Sugar Acids	1.7212	0.019955	0.001799
Citramalic Acid	Dicarboxylic Acids	1.6452	0.021471	0.001837
FA 14:0	Saturated Fatty Acids	1.563	0.021471	0.0022468
Cer d40:2	Unsaturated Ceramides	1.4075	0.021471	0.002369
Threonine	Amino Acids	0.68957	0.021533	0.0023896
2,3-Dihydroxybutanoic Acid	Sugar Acids	0.56986	0.021566	0.0022734
Phenylalanine	Aromatic Amino Acids	0.82718	0.022098	0.002455
Canrenone	Steroid	0.53944	0.022098	0.0025844
3-Hydroxybutyric acid	Hydroxybutyrates	2.7853	0.023245	0.0022229
TG 46:3	Unsaturated Triglycerides	0.50138	0.024382	0.0029347
SM d42:2	Sphingomyelins	1.3379	0.024465	0.0029545
Indole-3-carboxaldehyde	Indoles	0.67316	0.024832	0.0030554
TG 57:2	Unsaturated Triglycerides	0.64429	0.025268	0.003083
Pyruvic acid	Carboxylic Acids	1.8856	0.032598	0.0035975
SM d36:3	Sphingomyelins	0.72675	0.032598	0.0039276
4-Acetylbutyric acid	Adipates	0.67028	0.035547	0.0040286
Cer d40:1	Unsaturated Ceramides	1.282	0.035828	0.0044308
TG 62:4	Unsaturated Triglycerides	0.69618	0.035828	0.0047119
Ribonic Acid	Glutarates	1.5127	0.037173	0.0048206

Inositol-4-Monophosphate	Inositol Phosphates	0.75597	0.037382	0.0050257
TG 60:5	Unsaturated Triglycerides	0.71859	0.038029	0.0050994
Isoleucine	Branched Amino Acids	0.76345	0.038483	0.0052252
TG 46:5	Unsaturated Triglycerides	0.70891	0.038664	0.0049724
Lactosylceramide d18:1	Lactosyl Ceramide	1.3825	0.039516	0.0054443
PC 38:7	Phosphatidylethanolamines	0.72331	0.039825	0.0056433
TG 64:3	Unsaturated Triglycerides	0.74952	0.040082	0.0051105
LPC O-16:0	Lysophosphatidylcholine	1.5051	0.040854	0.0058803
Ethanolamine	Ethanolamines	1.2598	0.040854	0.0060548
TG 56:0	Saturated Triglycerides	0.74476	0.040854	0.0059091
PC 34:3	Phosphatidylcholine	0.71916	0.041441	0.0062682
3-Methylhistidine	Histidine	0.31949	0.041441	0.006094
TG 56:1	Unsaturated Triglycerides	0.39071	0.043122	0.0065553
TG 54:0	Saturated Triglycerides	0.62508	0.043428	0.0067656
Choline	Ethanolamines	1.411	0.043686	0.0061232
Cer d34:2	Unsaturated Ceramides	1.2055	0.045997	0.0072871
TG 44:2	Unsaturated Triglycerides	0.41224	0.047585	0.0075854
Lactamide	Amides	2.1543	0.047615	0.0065618
PE 36:2	Phosphatidylethanolamines	0.75952	0.04832	0.0072815
5-Hydroxymethylcytosine	Pyrimidine	0.6565	0.049225	0.0079814
TG 60:1	Unsaturated Triglycerides	0.50021	0.049225	0.0081749
Arabitol	Sugar Alcohol	1.1711	0.051924	0.008802
Ornithine	Basic Amino Acids	0.70016	0.051924	0.0082201
TG 58:1	Unsaturated Triglycerides	0.47291	0.051924	0.0088563
TG 57:1	Unsaturated Triglycerides	0.63338	0.052697	0.0091532
TG 47:2	Unsaturated Triglycerides	0.58704	0.052697	0.009184
TG 58:4	Unsaturated Triglycerides	0.71097	0.05292	0.0093097
Creatinine	Imidazoles	0.81286	0.053308	0.0093815
Phenylacetylglycine	Dipeptides	0.58673	0.053308	0.0093414
TG 45:1	Unsaturated Triglycerides	0.45352	0.053364	0.0092744
TG 42:2	Unsaturated Triglycerides	0.39772	0.053364	0.0096214
SM d43:2	Sphingomyelins	1.3957	0.05347	0.0098914
TG 50:6	Unsaturated Triglycerides	0.69975	0.05347	0.0098154
TG 55:1	Unsaturated Triglycerides	0.55863	0.053794	0.0097392
CAR 14:1	Carnitine	1.686	0.055306	0.010474
Piperine	Polyunsaturated Alkamides	0.28225	0.055306	0.010255
PC 42:6	Phosphatidylcholines	0.66338	0.056759	0.010627
4-Hydroxyhippuric acid	Hippurates	0.67826	0.057844	0.010821
PC 40:6	Phosphatidylcholines	0.84222	0.061047	0.012004
TG 62:3	Unsaturated Triglycerides	0.67907	0.061047	0.011784
TG 48:4	Unsaturated Triglycerides	0.56497	0.061047	0.012051
TG 60:4	Unsaturated Triglycerides	0.59177	0.061511	0.012225
Aconitic Acid	Succinates	2.2715	0.063219	0.0099351
Cer d42:1	Unsaturated Ceramides	1.2204	0.063219	0.012738
Cysteine	Sulfur Amino Acids	1.4085	0.064226	0.012582
TG 42:3	Unsaturated Triglycerides	0.45987	0.064923	0.013125
N-Methylvaline	Branched Amino Acids	0.78889	0.066051	0.013885
gamma-Glutamylleucine	Dipeptides	0.75446	0.066051	0.013663

TG 48:3	Unsaturated Triglycerides	0.59209	0.066051	0.013898
TG 46:2	Unsaturated Triglycerides	0.47971	0.066051	0.013853
Leucine	Branched Amino Acids	0.76988	0.066432	0.013949
PC 36:6	Phosphatidylethanolamines	0.70866	0.066432	0.014186
PC 38:6	Phosphatidylethanolamines	0.80739	0.067752	0.01447
Quinolone	Hydroxyquinolines	2.2462	0.071746	0.014157
TG 48:5	Unsaturated Triglycerides	0.6636	0.071746	0.015682
PE P-40:5	Phosphatidylethanolamines	1.3202	0.072109	0.01565
TG 56:2	Unsaturated Triglycerides	0.55435	0.073052	0.016169
SM d44:2	Sphingomyelins	1.2742	0.073249	0.016496
10-Hydroxydecanoic acid	Saturated Fatty Acids	0.51411	0.073249	0.015655
Acetylcarnitine	Carnitine	1.289	0.073283	0.016741
TG 58:2	Unsaturated Triglycerides	0.54647	0.073283	0.016829
Taurocholic acid	Taurine	0.26118	0.073283	0.016704
TG 60:3	Unsaturated Triglycerides	0.5902	0.076333	0.017607
TG 53:0	Saturated Triglycerides	0.69101	0.078977	0.018305
PE 34:2	Phosphatidylethanolamines	0.68127	0.08005	0.018553
TG 45:0	Saturated Triglycerides	0.50201	0.080355	0.016738
Threonic Acid	Carboxylic Acids	0.70598	0.081449	0.019132
TG 64:2	Unsaturated Triglycerides	0.75451	0.082143	0.019015
LPC 16:0	Saturated LPC	1.4178	0.08298	0.019032
Histidine	Cyclic Amino Acids	0.78943	0.08298	0.020183
PE 38:6	Phosphatidylethanolamines	0.61346	0.08298	0.019249
Glycerol-3-Galactoside	Sugar Alcohol	0.78002	0.084157	0.019529
TG 44:1	Unsaturated Triglycerides	0.33049	0.084861	0.020591
TG 44:0	Saturated Triglycerides	0.28817	0.08554	0.020627
SM d34:1	Sphingomyelins	1.1778	0.085696	0.020634
TG 42:0	Saturated Triglycerides	0.25815	0.085696	0.020738
PC 38:4	Phosphatidylethanolamines	0.87227	0.086678	0.02166
N8-Acetylspermidine	Spermidine	1.405	0.090285	0.022825
Allantoin	Hydantoins	0.77843	0.090911	0.023428
TG 62:1	Unsaturated Triglycerides	0.65493	0.090911	0.023271
Lactic Acid	Hydroxybutyrates	1.6265	0.091531	0.023927
LPC 18:0	Saturated LPC	1.4235	0.091531	0.023856
PE O-38:5	Phosphatidylethanolamines	1.2954	0.091531	0.023792
TG 58:3	Unsaturated Triglycerides	0.60576	0.091531	0.023668
1-Methylnicotinamide	Pyridines	0.1484	0.09267	0.021318
N-Methylalanine	Dipeptides	1.5189	0.094783	0.024901
TG 48:2	Unsaturated Triglycerides	0.64105	0.094783	0.025045
TG 60:2	Unsaturated Triglycerides	0.57778	0.094783	0.02557
TG 42:1	Unsaturated Triglycerides	0.2822	0.094783	0.025727
Sophorose	Disaccharides	1.5814	0.097388	0.023399
Valine	Branched Amino Acids	0.80343	0.098654	0.027212
Methionine Sulfoxide	Sulfur Amino Acids	0.77731	0.098654	0.027156
PE P-40:7	Phosphatidylethanolamines	0.79777	0.098841	0.026856
Phloroglucinol	Phenols	1.4247	0.099884	0.026457
P-Toluenesulfonic acid	Polystyrenes	0.46157	0.10142	0.026968
GlcCer d42:2	Glycosphingolipid	1.3458	0.10234	0.027299

LPC 20:0	Saturated LPC	1.3048	0.10415	0.029796
TG 50:5	Unsaturated Triglycerides	0.6744	0.10415	0.029888
TG 46:1	Unsaturated Triglycerides	0.42833	0.10415	0.030725
Glycocholate	Chenodeoxycholic Acid	0.522	0.10788	0.031133
TG 58:0	Saturated Triglycerides	0.82917	0.10829	0.032506
TG 47:1	Unsaturated Triglycerides	0.54315	0.11214	0.033756
TG 52:0	Saturated Triglycerides	0.61071	0.11526	0.033959
3-(3-Hydroxyphenyl)propionic Acid	Carboxylic Acids	0.60326	0.11526	0.033756
PC 31:1	Phosphatidylethanolamines	0.70986	0.11951	0.035375
TG 47:0	Saturated Triglycerides	0.56843	0.12033	0.034554
Histamine	Biogenic Monoamines	1.5249	0.12247	0.036615
CAR 10:0	Carnitine	1.8909	0.12254	0.036972
TG 62:2	Unsaturated Triglycerides	0.68665	0.12254	0.036972
5'-Methylthioadenosine	Adenosine	0.7985	0.13116	0.038678
TG 54:1	Unsaturated Triglycerides	0.40997	0.13163	0.039151
TG 40:0	Saturated Triglycerides	0.3011	0.13178	0.039646
TG 60:6	Unsaturated Triglycerides	0.72232	0.13249	0.039913
4-Pyridoxic acid	Pyridines	0.33111	0.13884	0.042597
PC P-44:5	Phosphatidylethanolamines	1.2935	0.14103	0.042816
TG 40:1	Unsaturated Triglycerides	0.34432	0.14192	0.043432
Glutamic acid	Acidic Amino Acids	1.4103	0.14353	0.044584
LPC 20:2	Unsaturated LPC	1.3889	0.14432	0.044993
Cer d33:1	Unsaturated Ceramides	1.155	0.14432	0.045316
Nudifloramide	Pyridines	0.57827	0.14432	0.045971
PE 36:1	Phosphatidylethanolamines	0.81628	0.1445	0.046514
PE 36:4	Phosphatidylethanolamines	0.71772	0.1445	0.046826
Urocanic Acid	Acrylates	0.42485	0.1445	0.043432
TG 49:3	Unsaturated Triglycerides	0.77176	0.14659	0.047286
CAR 8:0	Carnitine	1.6333	0.14973	0.048836
LPE 18:2	Unsaturated LPC	0.80024	0.14973	0.047749
LPC P-16:0	Saturated LPC	1.412	0.15166	0.049983

Table S2. Metabolites associated with MTX therapy. Phosphatidylcholine (PC); triglycerides (TG); fatty acid (FA); phosphatidylethanolamine (PE); ceramides (Cer); sphingomyelin (SM); carnitine (CAR).

Baseline V. Week16

Compound	Class	FC	q value	p value
TG 44:0	Saturated Triglycerides	2.3958	0.092056	0.0023989
TG 46:2	Unsaturated Triglycerides	2.1341	0.092056	0.0028469
TG 46:1	Unsaturated Triglycerides	2.0986	0.092056	0.0022139
TG 42:0	Saturated Triglycerides	2.0916	0.092056	0.0070592
TG 42:1	Unsaturated Triglycerides	2.0915	0.092056	0.0042266
TG 56:1	Unsaturated Triglycerides	2.0803	0.092056	0.0037114
TG 44:1	Unsaturated Triglycerides	2.0603	0.092056	0.0016353
TG 44:2	Unsaturated Triglycerides	2.0039	0.092056	0.0031098
TG 42:3	Unsaturated Triglycerides	1.9323	0.092056	0.00088467
TG 48:3	Unsaturated Triglycerides	1.8165	0.092056	0.0021025
TG 52:0	Saturated Triglycerides	1.7765	0.092056	0.0035328
TG 46:3	Unsaturated Triglycerides	1.7762	0.092056	0.0012508
TG 54:0	Saturated Triglycerides	1.7188	0.092056	0.0038774
TG 56:2	Unsaturated Triglycerides	1.6464	0.092056	0.0036265
TG 48:2	Unsaturated Triglycerides	1.6087	0.092056	0.0014044
TG 47:2	Unsaturated Triglycerides	1.5807	0.092056	0.0006393
TG 45:1	Unsaturated Triglycerides	1.5532	0.092056	0.00015114
TG 51:0	Saturated Triglycerides	1.539	0.092056	0.0039449
TG 59:2	Unsaturated Triglycerides	1.5258	0.092056	0.0027912
TG 47:1	Unsaturated Triglycerides	1.5249	0.092056	0.00053116
TG 49:0	Saturated Triglycerides	1.4717	0.092056	0.0038425
TG 48:1	Unsaturated Triglycerides	1.4299	0.092056	0.0040456
TG 49:2	Unsaturated Triglycerides	1.3976	0.092056	0.0039749
TG 47:0	Saturated Triglycerides	1.3943	0.092056	0.0023622
TG 49:3	Unsaturated Triglycerides	1.394	0.092056	0.0037036
TG 45:0	Saturated Triglycerides	1.3606	0.092056	0.0015816
TG 56:3	Unsaturated Triglycerides	1.3594	0.092056	0.0042737
TG 55:2	Unsaturated Triglycerides	1.3285	0.092056	0.0024901
TG 54:2	Unsaturated Triglycerides	1.306	0.092056	0.0036085
Oleic Acid	Unsaturated Fatty Acids	0.58722	0.092056	0.0034458
FA 22:2	Unsaturated Fatty Acids	0.5811	0.092056	0.00032173
TG 46:0	Saturated Triglycerides	1.8112	0.092351	0.004876
TG 54:1	Unsaturated Triglycerides	1.8016	0.092351	0.0047161
TG 60:1	Unsaturated Triglycerides	1.7225	0.092351	0.0050052
TG 56:0	Saturated Triglycerides	1.5204	0.092351	0.0048823
TG 57:2	Unsaturated Triglycerides	1.3611	0.092351	0.0051306
TG 40:1	Unsaturated Triglycerides	1.5872	0.096741	0.0055238
FA 17:1	Unsaturated Fatty Acids	0.54271	0.10624	0.00623
TG 42:2	Unsaturated Triglycerides	2.1488	0.11157	0.0070592
TG 58:1	Unsaturated Triglycerides	1.817	0.11157	0.0067734
TG 55:1	Unsaturated Triglycerides	1.4578	0.11157	0.0069148
2-Amino-3-Methoxybenzoic Acid	Aminobenzoates	0.3491	0.11239	0.0072844
TG 57:1	Unsaturated Triglycerides	1.5406	0.11342	0.0089949

TG 53:0	Saturated Triglycerides	1.4272	0.11342	0.0085777
TG 49:1	Unsaturated Triglycerides	1.3527	0.11342	0.0091019
TG 52:1	Unsaturated Triglycerides	1.3514	0.11342	0.0077572
TG 58:4	Unsaturated Triglycerides	1.3421	0.11342	0.0085074
TG 62:4	Unsaturated Triglycerides	1.3061	0.11342	0.00818
PE 36:2	Phosphatidylethanolamines	1.176	0.11342	0.0087321
Cer d34:1	Unsaturated Ceramides	0.86931	0.11342	0.010168
Cer d33:1	Unsaturated Ceramides	0.85115	0.11342	0.0087224
FA 18:3	Unsaturated Fatty Acids	0.55334	0.11342	0.0088893
TG 50:0	Saturated Triglycerides	1.585	0.1156	0.0097087
TG 46:4	Unsaturated Triglycerides	1.5561	0.1156	0.0098762
Cer d34:2	Unsaturated Ceramides	0.87625	0.1156	0.010168
FA 18:2	Unsaturated Fatty Acids	0.64154	0.1156	0.0099749
FA 16:1	Unsaturated Fatty Acids	0.50124	0.1156	0.010062
TG 55:3	Unsaturated Triglycerides	1.2102	0.11827	0.010586
Acetominophen	Acetanilides	0.30246	0.11884	0.01082
PC 32:0	Saturated PC	0.89428	0.12585	0.011684
Dehydroisoandrosterone Sulfate	Dehydroepiandrosterone	0.619	0.12585	0.011847
TG 48:4	Unsaturated Triglycerides	1.7526	0.13166	0.0128
Coniferylaldehyde	Hydroxybenzoates	1.7306	0.13166	0.012766
TG 53:1	Unsaturated Triglycerides	1.4091	0.13243	0.013079
TG 50:5	Unsaturated Triglycerides	1.3718	0.13846	0.013996
TG 50:2	Unsaturated Triglycerides	1.2384	0.13846	0.014103
TG 50:4	Unsaturated Triglycerides	1.3978	0.14218	0.014832
TG 58:0	Saturated Triglycerides	1.3398	0.14218	0.014985
Succinic Acid	Succinates	0.7645	0.14218	0.015139
SM d34:1	Sphingomyelins	0.91298	0.14272	0.015417
TG 62:3	Unsaturated Triglycerides	1.3886	0.14579	0.015974
FA 14:1	Unsaturated Fatty Acids	0.54941	0.14579	0.016199
TG 60:2	Unsaturated Triglycerides	1.6606	0.1547	0.017427
TG 48:0	Saturated Triglycerides	1.4988	0.17117	0.019548
TG 51:1	Unsaturated Triglycerides	1.3376	0.17314	0.020039
TG 58:2	Unsaturated Triglycerides	1.7279	0.17491	0.020669
1,3,7-Trimethyluric acid	Xanthines	1.5808	0.17491	0.02188
TG 60:3	Unsaturated Triglycerides	1.5422	0.17491	0.020912
TG 50:3	Unsaturated Triglycerides	1.2894	0.17491	0.022133
TG 56:4	Unsaturated Triglycerides	1.2344	0.17491	0.021721
TG 54:3	Unsaturated Triglycerides	1.1736	0.17491	0.021722
SM 42:1;2O	Sphingomyelins	0.89673	0.17491	0.021246
TG 48:5	Unsaturated Triglycerides	1.4966	0.17783	0.022986
Homogentisic acid	Carbocyclic Acids	1.4852	0.17783	0.02307
FA 17:0	Saturated Fatty Acids	0.76296	0.17783	0.023326
TG 62:2	Unsaturated Triglycerides	1.4538	0.1809	0.024227
TG 51:5	Unsaturated Triglycerides	1.2072	0.1809	0.024523
TG 50:1	Unsaturated Triglycerides	1.179	0.1809	0.024566
TG 50:6	Unsaturated Triglycerides	1.4258	0.19828	0.027234
TG 62:1	Unsaturated Triglycerides	1.5103	0.20451	0.028967
PE 36:1	Phosphatidylethanolamines	1.1333	0.20451	0.029351

Cer d34:0	Saturated Ceramides	0.81584	0.20451	0.029249
Heptadecanoic Acid	Saturated Fatty Acids	0.80075	0.20451	0.029245
TG 58:3	Unsaturated Triglycerides	1.5593	0.20576	0.030042
TG 55:4	Unsaturated Triglycerides	1.1756	0.20576	0.030166
Caffeine	Xanthine	1.9604	0.20666	0.030674
Tryptophan	Aromatic Amino Acids	1.2714	0.20666	0.030964
FA 15:1	Unsaturated Fatty Acids	0.71124	0.20666	0.031255
TG 60:4	Unsaturated Triglycerides	1.371	0.20957	0.032017
Glycocholate	Chenodeoxycholic Acid	1.5909	0.21491	0.033368
Acetaminophen Sulfate	Acetanilids	0.20726	0.21491	0.033496
TG 60:6	Unsaturated Triglycerides	1.1648	0.217	0.034158
TG 40:0	Saturated Triglycerides	1.7548	0.22222	0.035322
Phosphate	Polymers	0.79844	0.22283	0.035762
TG 59:3	Unsaturated Triglycerides	1.3231	0.22313	0.036407
SM d42:2	Sphingomyelins	0.88994	0.22313	0.036499
CAR 18:2	Carnitine	0.70177	0.24336	0.040184
3-(2-Hydroxyethyl)indole	Indoles	0.81471	0.24482	0.040803

Table S3. Chemometric enrichment analysis of metabolites associated with induction of RA.

Cluster name	P Value	Q Value	Fractional Change	X LOG P
Unsaturated Triglycerides	2.2E-20	1.2E-18	0.46067415	24.8615955
Saturated Triglycerides	4.3E-10	0.00000001	0.61538461	22.8636429
Unsaturated Ceramides	0.00000001	0.00000013	0.5625	15.5865
Unsaturated Fatty Acids	0.00000006	0.00000074	0.588235294	8.73633333
Carnitine	0.00000007	0.00000074	0.714285714	4.303
Hydroxybutyrates	0.00015	0.0014	0.666666667	-0.0542
Glutarates	0.00035	0.0028	0.5	-1.71433333
Sugar Acids	0.0011	0.0077	0.25	-1.7295
Sugar Alcohols	0.0014	0.0089	0.181818182	-3.62690909
Amino Acids, Branched-Chain	0.0021	0.012	-0.4	-1.4945
Amino Acids, Basic	0.0024	0.012	-0.333333333	-3.692
Carbocyclic Acids	0.0026	0.012	0	0.2665
Sphingomyelins	0.0029	0.013	0.068965517	14.756875
Sat. Lysophosphatidylcholines	0.0041	0.015	0.666666667	5.79766667
Succinates	0.0041	0.015	0.75	-0.72
Disaccharides	0.0062	0.022	0.285714286	-1.7295
Butyrates	0.013	0.041	0.333333333	-2.76375
Indoles	0.013	0.041	-0.333333333	0.8366
Phosphatidylethanolamines	0.015	0.043	-0.142857143	14.8576
Adenosine	0.024	0.067	0.5	-1.9
Ethanolamines	0.041	0.11	0.666666667	-1.14366667
Biogenic Monoamines	0.047	0.12	0.666666667	-0.4555

Table S4. Chemometric enrichment analysis of metabolites associated MTX Therapy.

Cluster Name	P Values	Q Value	Fractional Change	X LOG P
Unsaturated Sphingomyelins	2.2E-20	1.2E-18	-0.551724138	24.8616
Unsaturated Triglycerides	1.1E-16	3.1E-15	0.359550562	15.68639
Unsaturated Phosphatidylcholines	5.3E-14	9.8E-13	-0.174603175	15.65769
Saturated Triglycerides	1.3E-13	1.9E-12	0.692307692	14.8576
Unsaturated Ceramides	9.8E-13	1.1E-11	-0.625	22.86364
Unsaturated Fatty Acids	8E-12	7.4E-11	-0.705882353	7.46525
Unsat. Lysophosphatidylcholines	0.000000095	0.0000007	-0.285714286	15.8855
Cholesterol Esters	0.000000098	0.0000007	-0.555555556	8.736333
Plasmalogens	0.00000033	0.000002	-0.625	-3.62691
Phosphatidylinositols	0.0000078	0.000043	-0.857142857	6.2037
Saturated Fatty Acids	0.00027	0.0014	-0.333333333	16.15725
Dipeptides	0.00041	0.0019	-0.222222222	-1.7295
Sugar Alcohols	0.00052	0.0023	-0.272727273	15.42157
Hydroxybutyrates	0.00067	0.0027	-0.333333333	13.61457
Saturated Sphingomyelins	0.0014	0.0053	-0.333333333	4.303
Dicarboxylic Acids	0.0022	0.0079	-0.428571429	5.797667
Carnitine	0.0027	0.0089	-0.214285714	-2.62333
Succinates	0.0052	0.016	-0.75	-0.0542

Table S5. Metabolite levels displaying a statistically significant inverse correlation ($p < 0.05$) with DAS-28. Fatty Acid (FA); triglycerides (TG); lysophosphatidylethanolamides (LPE); phosphatidylcholine (PC, PC-O).

DAS-28				
**Metabolite**	**Pathway Effected**	**Metabolite Class**	**Spearman's rho**	**p value**
TG 54:0	FA Metabaolism	Sat. Triglycerides	-0.4897	0.0013
TG 52:0	FA Metabaolism	Sat. Triglycerides	-0.4589	0.0029
TG 53:0	FA Metabaolism	Sat. Triglycerides	-0.4553	0.0032
TG 56:0	FA Metabaolism	Sat. Triglycerides	-0.4482	0.0037
TG 54:1	FA Metabaolism	Unsat. Triglyceries	-0.4439	0.0041
TG 45:0	FA Metabaolism	Sat. Triglycerides	-0.4394	0.0046
TG 48:0	FA Metabaolism	Sat. Triglycerides	-0.4332	0.0052
TG 50:0	FA Metabaolism	Sat. Triglycerides	-0.4218	0.0067
TG 55:1	FA Metabaolism	Unsat. Triglyceries	-0.4152	0.0077
TG 49:0	FA Metabaolism	Sat. Triglycerides	-0.4146	0.0078
TG 51:0	FA Metabaolism	Sat. Triglycerides	-0.4083	0.0089
TG 47:0	FA Metabaolism	Sat. Triglycerides	-0.4066	0.0092
TG 52:1	FA Metabaolism	Unsat. Triglyceries	-0.4041	0.0097
TG 56:1	FA Metabaolism	Unsat. Triglyceries	-0.3961	0.0114
TG 46:0	FA Metabaolism	Sat. Triglycerides	-0.3932	0.0121
TG 50:1	FA Metabaolism	Unsat. Triglyceries	-0.3887	0.0132
LPE 22:6	FA Metabolism	Unsat. Lysolecithins	0.378	0.0162
TG 44:0	FA Metabaolism	Sat. Triglycerides	-0.3724	0.018
TG 47:1	FA Metabaolism	Unsat. Triglyceries	-0.3685	0.0193
TG 53:1	FA Metabaolism	Unsat. Triglyceries	-0.3583	0.0232
TG 57:1	FA Metabaolism	Unsat. Triglyceries	-0.3508	0.0264
TG 58:0	FA Metabaolism	Sat. Triglycerides	-0.3482	0.0277
PC 36:5	FA Metabolism	Phosphatidylcholines	0.3443	0.0296
Oxaloacetic acid	Citric Acid Cycle	Oxaloacetates	0.342	0.0308
TG 45:1	FA Metabaolism	Unsat. Triglyceries	-0.3403	0.0316
TG 46:1	FA Metabaolism	Unsat. Triglyceries	-0.3379	0.033
TG 44:1	FA Metabaolism	Unsat. Triglyceries	-0.3364	0.0338
TG 46:2	FA Metabaolism	Unsat. Triglyceries	-0.3351	0.0346
Hypoxanthine	Purine Biosynthesis	Hypoxanthines	-0.333	0.0357
PC 38:5	FA Metabolism	Phosphatidylcholines	0.3321	0.0363
Gluconolactone	Tricarboxylic Acid Cycle	Gluconates	-0.3291	0.0381
TG 54:2	FA Metabaolism	Unsat. Triglyceries	-0.3197	0.0443
PC O-38:8	FA Metabolism	Phosphatidylcholines	0.3182	0.0454
TG 56:2	FA Metabaolism	Unsat. Triglyceries	-0.3161	0.0469
Secoisolariciresinol	Gut Microflora	Lignan	0.3141	0.0484
TG 51:1	FA Metabaolism	Unsat. Triglyceries	-0.3139	0.0486

Table S6. Metabolites of interest ($p<0.05$) identified in volcano plots to display a correction towards healthy control following MTX therapy. Fatty acid (FA); ceramide (Cer); triglyceride (TG).

		Control V. Disease		Disease V. MTX	
**Compound**	**Metabolite Class**	**FC**	**p value**	**FC**	**p value**
Homogentisic Acid	Carbocyclic Acid	0.29183	1.03E-08	1.4852	0.043844
FA 22:2	Fatty Acids	2.1546	3.58E-06	0.5811	0.000907
FA 18:3	Fatty Acids	2.9349	1.77E-05	0.55334	0.020167
Cer d34:1	Ceramides	1.3169	0.001178	0.86931	0.0178
FA 16:1	Fatty Acids	2.7461	7.06E-05	0.50124	0.008705
FA 17:1	Fatty Acids	2.3176	8.34E-05	0.54271	0.006529
FA 18:2	Fatty Acids	1.944	0.00037	0.64154	0.015671
TG 59:2	Triglycerides	0.56996	0.000422	1.5258	0.013044
FA 14:1	Fatty Acids	2.1018	0.00233	0.54941	0.015211
Oleic Acid	Fatty Acids	2.7812	0.001082	0.58722	0.039712
TG 46:3	Triglycerides	0.50138	0.002935	1.7762	0.020033
TG 57:2	Triglycerides	0.64429	0.003159	1.3611	0.025562
TG 56:0	Triglycerides	0.74476	0.006116	1.4258	0.027048
TG 54:0	Triglycerides	0.62508	0.006769	1.7188	0.020947
Cer d34:2	Ceramides	1.2055	0.007311	0.87625	0.048301
TG 44:2	Triglycerides	0.41224	0.007637	2.0039	0.027111
TG 57:1	Triglycerides	0.63338	0.00917	1.5406	0.028867
TG 47:2	Triglycerides	0.58704	0.009189	1.5807	0.044277
TG 45:1	Triglycerides	0.45352	0.009694	1.5532	0.017762
TG 42:2	Triglycerides	0.39772	0.009718	2.1488	0.026871
TG 55:1	Triglycerides	0.55863	0.010045	1.4578	0.037719
TG 48:4	Triglycerides	0.56497	0.012059	1.7526	0.038033
TG 42:3	Triglycerides	0.45987	0.013325	1.9323	0.017557
TG 48:3	Triglycerides	0.59209	0.013964	1.8165	0.019513
TG 56:2	Triglycerides	0.55435	0.016234	1.6464	0.04153
TG 53:0	Triglycerides	0.69101	0.018404	1.4272	0.039637
TG 45:0	Triglycerides	0.50201	0.018973	1.3606	0.015729
TG 44:1	Triglycerides	0.33049	0.020953	2.0603	0.02451
TG 44:0	Triglycerides	0.28817	0.021253	2.3958	0.009258
TG 48:2	Triglycerides	0.64105	0.025628	1.6087	0.049873
TG 42:1	Triglycerides	0.2822	0.025623	2.0915	0.028198
TG 46:1	Triglycerides	0.42833	0.029613	2.0986	0.027198
TG 52:0	Triglycerides	0.61071	0.033974	1.7765	0.036032
TG 47:0	Triglycerides	0.56843	0.035839	1.3943	0.047854
Cer d33:1	Ceramides	1.155	0.0461	0.85115	0.028384

Table S7. ROC analysis of metabolite levels between responders and non-responders defined by δDAS-28(-1.2) after 16 weeks of MTX therapy. Phosphoethanolamines (PE-P); phosphatidylcholines (PC-P, PC-O, PC); lysophosphatidylcholines (LPC); fatty acid (FA); carnitine (CAR).

Compound	Metabolite Class	AUC	T-Test	Log2 FC
2-Amino-3-methoxybenzoic acid	Aminobenzoates	0.87879	0.003899	-4.5739
3-Methyladipic acid	Adipates	0.87879	0.003968	-0.69398
Nicotinamide	Pyridines	0.84848	0.021258	-1.6245
Mandelic acid	Carbocyclic Acids	0.84848	0.007704	-0.7225
Orthanilic acid	Sulfanilic Acids	0.84848	0.006789	-1.096
N-Acetyltryptophan	Indoles	0.83838	0.004472	-0.42204
Isoheptadecanoic Acid	Fatty Acids	0.82828	0.017848	0.8043
Stachydrine	Imino Acids	0.80808	0.01925	1.8515
Tyrosine	Aromatic Amino Acids	0.80808	0.030277	1.3871
Acetylenedicarboxylic acid	Alkynes	0.80808	0.019285	-0.31704
Aspartic Acid	Acidic Amino Acids	0.79798	0.019278	-0.52917
Acetominophen	Acetanilides	0.79798	0.015532	-5.2895
2-Hydroxyisobutyric acid	Hydroxybutyrates	0.78788	0.022503	-0.7137
Hypoxanthine	Hypoxanthines	0.78788	0.02615	-1.2261
6-Hydroxycaproic acid	Adipates	0.78788	0.026694	-0.43733
1,5-Anhydrosorbitol	Hexoses	0.78788	0.017151	0.4026
Pyroglutamic Acid	Dipeptides	0.78788	0.028985	-0.28926
Erythronolactone	Butyrates	0.78788	0.032892	-0.72951
PE P-40:4	Phosphoethanolamines	0.78788	0.044687	-0.57956
PC P-42:2	Phosphatidylcholines	0.78788	0.020008	0.59889
Tartaric Acid	Dicarboxylic Acids	0.77778	0.034459	-0.53226
N-Methylalanine	Dipeptides	0.77778	0.018695	-0.63236
Methylacetate	Acetates	0.77778	0.026674	-0.66585
Inosine	Purine Nucelosides	0.77778	0.043939	-1.2844
PC P-34:0	Phosphotidylcholines	0.77778	0.065236	0.37313
Valine	Branched-chain Amino Acids	0.76768	0.028678	-0.43714
Glycolic acid	Glycolates	0.76768	0.067438	-0.38357
Choline	Ethanolamines	0.76768	0.017433	-0.54516
Glutamic acid	Acidic Amino Acids	0.76768	0.021183	-0.64906
LPC 22:5	Lysophosphatidylcholines	0.76768	0.13774	-0.46771
PC P-42:5	Phosphatidylcholines	0.76768	0.037623	0.51995
Itaconic Acid	Succinates	0.75758	0.088784	0.24652
indole-3-propionic acid	Indoles	0.75758	0.04064	1.2257
conduritol-beta-epoxide	Cyclitols	0.75758	0.024536	1.1941
N-Methylvaline	Branched-chain Amino Acids	0.75758	0.035791	-0.39955
alpha-Hydroxyhippuric acid	Hippurates	0.75758	0.096124	-0.84907
Histidine	Cyclic Amino Acids	0.75758	0.019572	-0.43478
Methylsuccinic acid	Succinates	0.75758	0.017819	-0.52678
N,N-Dimethylformamide	Formamides	0.75758	0.037487	-0.31302
FA 22:5	Fatty Acids	0.75758	0.14831	-0.92727
PC 36:4	Phosphatidylcholines	0.75758	0.08112	0.26191
PC O-44:5	Phosphatidylcholines	0.75758	0.040682	0.58488
CAR 12:0	Carnitine	0.75758	0.0748	-0.51045

PC P-44:5 or PC O-44:6	Phosphatidylcholines	0.75758	0.039599	0.54814
